# Mapping the global election landscape on social media in 2024

**DOI:** 10.1371/journal.pone.0316271

**Published:** 2025-02-05

**Authors:** Giulio Pecile, Niccolò Di Marco, Matteo Cinelli, Walter Quattrociocchi

**Affiliations:** Department of Computer Science, Sapienza University of Rome, Rome, Italy; University of Salerno, ITALY

## Abstract

In 2024, a significant portion of the global population will participate in elections, creating an opportunity to analyze how information spreads and how users behave on social media. This study examines the media landscape on Facebook by analyzing posts from political parties and major news outlets in Europe, Mexico, and India. By identifying key topics and measuring public engagement, we uncover patterns in political discourse. Using Principal Component Analysis, we explore how these topics are related and distinguish trends in audience interaction. Our findings show how certain topics resonate differently across political groups, providing insights into the relationship between media content, political ideology, and user engagement during elections.

## Introduction

The advent and proliferation of social media platforms have fundamentally altered the information landscape and how we interact online [1], transforming these platforms into essential tools for getting information [2, 3], entertainment [4, 5], and personal communication [6]. As social media platforms have become embedded in our daily lives, they have blended entertainment-driven business models with complex social dynamics [7], raising concerns about their impact, particularly regarding polarization [8–11], where tailored content can reinforce divisions [12] and facilitate the spread of misinformation [13–15].

Thus, social media platforms have revealed to be critical arenas which saw an explosion of information and misinformation for global events, including the COVID-19 pandemic [16, 17], political events [18–20] and discussions on emerging technologies such as large language models [21, 22] and their implications for communication and automation. Moreover, these platforms play a pivotal role in consolidating biased views of the political landscape, potentially influencing public opinion and voter behavior during elections [19, 23] through the rapid dissemination and amplification of political content [24].

Research on how platform-specific effects influence social dynamics has highlighted several challenges [10, 12, 25–27]. Indeed, these interactions often exhibit persistent patterns despite different platforms, topics, and contexts, suggesting underlying consistencies in online human behavior [1]. Online users show the tendency to selectively expose to information [28, 29], preferring content that aligns with their pre-existing beliefs while avoiding contrary evidence [30, 31]. This behavior may fosters the emergence of homophilic communities, also know as echo-chambers [8], which significantly influence belief formation and communication methods [12], especially during delicate periods such as elections.

As the 2024 elections approach, with a substantial global population expected to vote, Facebook is a crucial platform for electoral campaigning. This period offers an invaluable opportunity to comprehensively analyze how different countries use social media for news consumption, public debate, and electoral influence.

In this study, we analyze content from news agencies and political parties on Facebook to see how it affects user interactions (including reactions, comments and shares) and examine variations between countries and the political leanings of parties. Using Principal Component Analysis, our analysis shows the main trends of the public discourse within the social media ecosystem, allowing us to identify patterns of both correlation and differences among key themes based on geographic and political distinctions.

Our comparative study across multiple countries aims to provide a multilateral understanding of how discussions on social media reflect electoral dynamics in a pivotal election year. Additionally, this paper explores how different countries, and thus socio-cultural factors, shape user interactions and interests on social media, contributing to the global dialogue on democracy and public discourse.

The structure of the paper is the following: We first assess the level of engagement on social media across various countries, then we explore the controversy surrounding discussions on diverse topics and, finally, we categorize the information and topics based on political leanings, providing a comprehensive overview of the digital electoral landscape in 2024.

## Materials and methods

### Dataset

We focus our analysis on a subset of countries having elections in 2024, excluding the ones where the use of Facebook is not prevalent or available to the population (e.g. Russia, where the site is blocked). Among the remaining countries, we choose a subset of them to make the project manageable, prioritizing the biggest democracies. We manually compile detailed lists of Facebook pages from each country in the European Union, as well as the UK, India, and Mexico. These lists include pages linked to the websites of political parties represented in national parliaments and major news agencies in each country. We also include major American news agencies and public Facebook pages of influential politicians, identified using the YouGov public survey platform [32]. Our dataset includes 508 news agencies and 336 political parties across 31 countries, capturing a total of approximately 4.2 million and 176 thousand posts between September 1, 2023, and May 1, 2024. For each post, we record the page name, posting time, textual content, and aggregated metrics like the number of reactions and comments. The data collection process is performed through CrowdTangle [33], a public insights tool owned and operated by Facebook, and was analyzed in compliance with its terms and conditions. A list of the pages used in the analysis is found in S1 Table.

### News outlet and parties labeling

In our study, we analyze Facebook pages from various countries that provide diverse perspectives on current events, ranging from the progressive left to the conservative right. We manually check political parties’ ideological leanings, since they are often clear and aligned with international coalitions, such as those within the European Parliament. Unlike political parties, news outlets usually have less explicit political alignments. To classify these, we use ratings from Media Bias/Fact Check (MBFC), an independent website that evaluates news agencies based on factuality and political bias. While MBFC recognizes a wide range of political leanings, for analytical simplicity, we consolidate these into three primary categories: ‘Left,’ ‘Center,’ and ‘Right.’

### Process of topic modelling

We implement a two-step process to assess the topics discussed in each post. We process each country separately since each one has posts in their primary language. For each country, we select a random sample of 50,000 posts made by news organizations. We then clean the text of these posts by removing links, hashtags, and non-alphanumeric characters, such as emojis. Subsequently, we apply the BERTopic clustering algorithm to perform topic clustering for each country. The resulting clusters are labelled with overarching themes such as ‘economy,’ ‘crime,’ ‘entertainment,’ and ongoing conflicts in Ukraine and the Middle East (identified as ‘mena’ in the analysis). Notice that Finland and Sweden do not produce any discernible topics during the clustering phase. To infer the topic of all posts, we compute the term frequency-inverse document frequency (tf-idf) values of the words from the posts within each cluster, treating the concatenation of all posts from each macro-topic as a single document. This allows us to identify the macro-topic that most closely aligned with the textual content of each post based on the tf-idf scores of the words. At the end of this process, we assess the topic of more than 3.5 million and 145 thousand posts from, respectively, news agencies and political parties of 31 countries.

**Fig 1:**
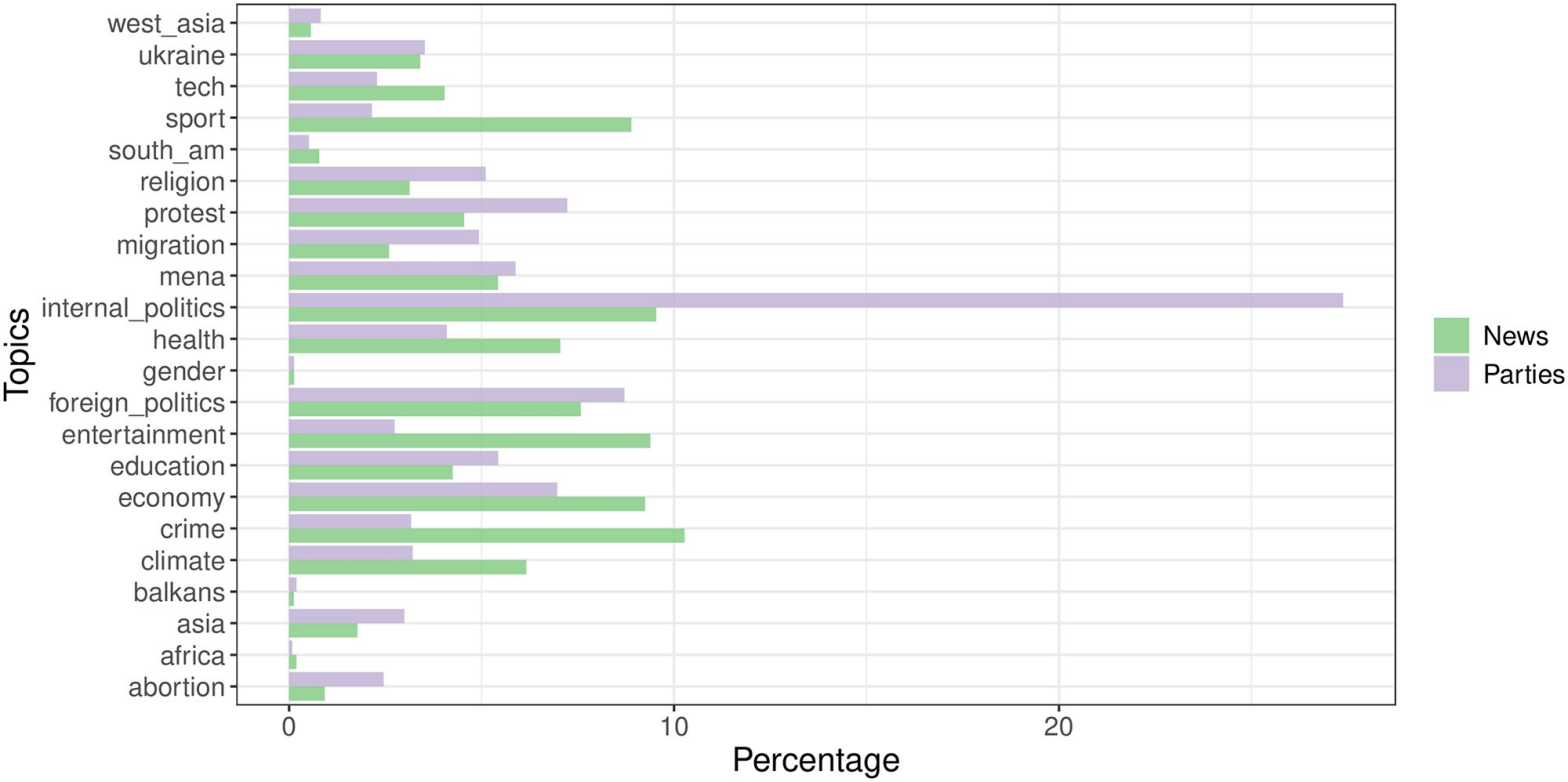
Number of posts within each topic for news outlets and political parties. The plurality of posts from political parties is about internal politics, while news agencies cover topics more evenly.

Fig 1 presents the share of posts discussing each topic. As shown, political parties predominantly post about internal politics, whereas ‘lighter’ topics such as entertainment or sports garner significantly more attention from news outlets.

In S1 Fig we provide a time series snapshot of the most covered topics by, respectively, American news organizations and politicians, grouped by political leaning. We can see that, barring a few spikes, most notably the one in October for posts covering the mena region, there is no clear trend indicating a change in interest over time. Moreover, the proportion of interest given to topics by either leaning does not change over time. Similarly, S2 Fig shows the average number of likes received by posts from a certain topic. While we can observe variations in the period of analysis, it is not possible to discern any distinctive trend.

Finally, to check the robustness of our method, we compare the results obtained by BERTopic with an LDA model on the news dataset from the UK. We present the overlap between the two classifications in S3 Fig, where we can see that the clustering structure of the first model is not reflected in the second one. Since there is no ground truth value for the topic of posts, we can only empirically assess the quality of the models based on observation. To do that, in S2 Table we provide a small sample of the two classifications, where it is evident that BERTopic outperforms the LDA model.

### Principal component analysis

Principal Component Analysis (PCA) [34] is a method for linear dimensionality reduction, widely used to summarize and visualize the information of datasets containing a large number of individuals or observations in lower dimensions containing the greater possible variance [35, 36].

In this work, we mostly use biplots [37] and methods to visualize the overall relation of individuals or variables. Therefore we briefly recall some aspects used in this work.

Consider a matrix *M* of dimension *n* × *m* in which rows are individuals and columns are variables measured on each individual. The entry *M*_*i*,*j*_ contains the *j*–th variable of individual *i*.

After applying PCA to the columns of *M* (each column is a variable) it is common to represent the results in a biplot, a two-dimensional space containing the larger possible variance for both variables and individuals, even if their coordinates are not constructed onto the same space.

Here, we summarize their properties:

- the distance between the variables and the origin is a measure of how well that variable is explained in two dimensions;
- points close to the origin of the axis indicate that those individuals have values close to variables mean values;
- if *i* and *j* are variables, we have cos *θ*_*ij*_ = *r*_*ij*_, i.e. the cos of the angle between two variables is equal to their correlation. Therefore, positively correlated variables are grouped together, while negatively correlated variables are positioned on opposite quadrants;
- individuals with similar values of variables are grouped together;
- an individual that is on the same (opposite) side of a certain variable has a high (low) value for that variable.

Note that variables and individuals could also be represented separately, keeping the properties listed above. In this work, we use the packages *FactoMineR* [38] and *factoextra* [39] to apply PCA and construct the relative biplots. In particular, we represent each individual as a black point, while variables are represented using red vectors starting from the origin.

## Results and discussion

### Engagement across countries

**Fig 2:**
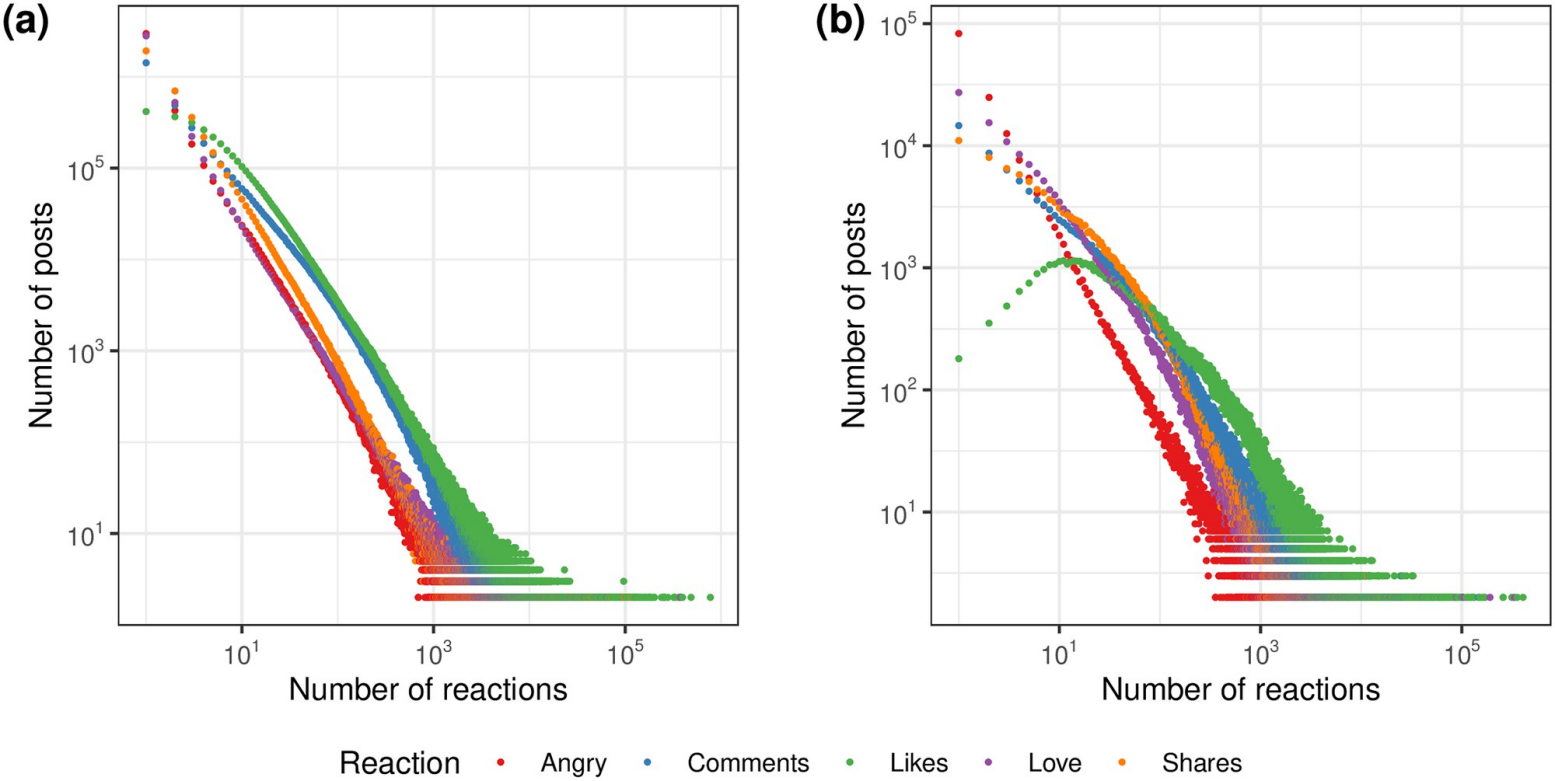
Distribution of reactions. (*a*) News outlets dataset and (*b*) Parties dataset. Both cases present a general trend of scale-free behavior. A notable exception from the general trend is the distribution of likes received by political parties.

We start the analysis by exploring the distribution of user engagement, which we measure through the reactions received by posts. Fig 2 illustrates the distribution of these interactions for both news outlets and political parties.

We observe that reactions across different types of content exhibit similar distributions. This pattern holds for both news agencies and political parties, as illustrated in Fig 2(*a*) and 2(*b*). However, a significant deviation occurs in the likes received by political parties, where the mode of the distribution is approximately 10. This indicates a reduced frequency of posts that receive very few likes for these entities. This behavior may be the effect of echo chamber phenomena, which would entail strong levels of homophily. Other explanations include the development of parasocial relationships with politicians, who are then identified as friends, and the presence of automated bots that add fake interactions to the count. Moreover, consistent with prior research [1], heavy tails characterize all the observed distributions, suggesting that while most posts receive relatively few reactions, a small number accumulates a disproportionately large number.

Interestingly, the number of reactions received by posts from political parties and news agencies falls within similar orders of magnitude, with tails ranging between 10^3^ and 10^5^ reactions. Engagement distribution patterns for each country are displayed in S4 Fig for news agencies and S5 Fig for political parties, exhibiting the same consistent patterns.

### Controversial topics across countries

**Fig 3:**
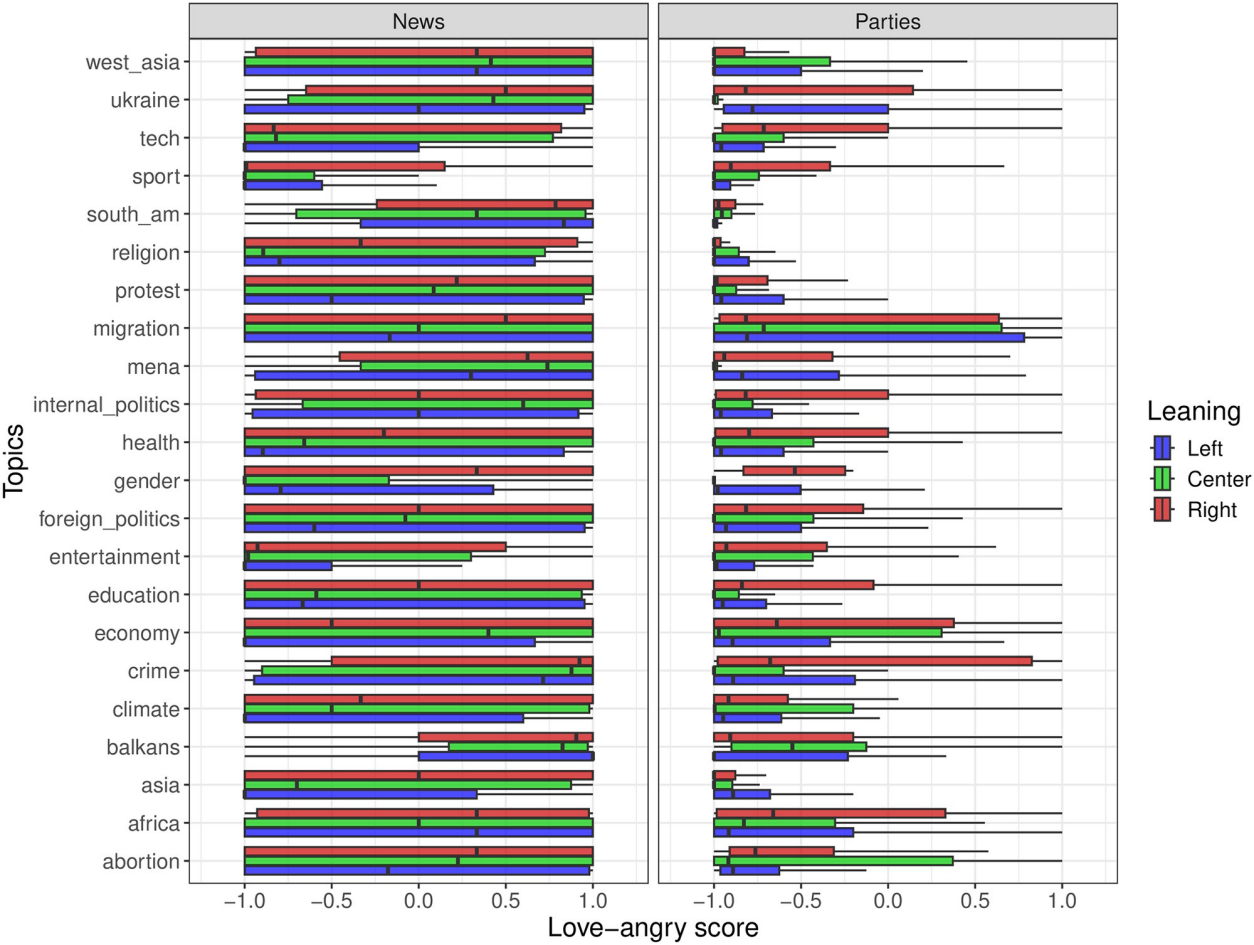
Boxplots of love-angry scores received for each topic. News pages (on the left) generally meet higher values of this metric. Political parties (on the right) find higher values of the score only for selected topics.

In this section, we investigate which topics capture more contentious debate by utilizing a metric derived from the number of ‘love’ and ‘angry’ reactions each post receives. Specifically, we define the *love-angry* score [4] for post *i*, denoted as *LA*(*i*), using the following equation:

$$\begin{array}{c} LA\left(i\right)=\frac{a_{i}-l_{i}}{a_{i}+l_{i}}, \end{array}$$

where *a*_*i*_ represents the number of angry reactions and *l*_*i*_ the number of love reactions received by post *i*. This metric serves to quantify the degree of divisiveness elicited by each post. In particular, the score takes values in [−1, +1], with −1 (+1) corresponding to posts that have received only love (angry) reactions. Fig 3 displays the distribution of love-angry scores of news agencies and political parties grouped according to their political leaning.

The results show that news agencies often receive higher ‘love-angry’ scores on their posts than political parties. This suggests news agencies reach a wider audience, attracting diverse reactions. On the other hand, posts from political parties generally see lower scores, indicating they might be engaging a more specific group of followers that are likely to share similar views and which could imply less debate and more agreement, typical of an echo chamber effect [8, 12].

### Topics in each country

In this section, we aim to highlight the most discussed topics in each country, using the total number of interactions as a proxy for their relative perceived importance. To avoid spurious results and focus on a meaningful subset of the data, from this point onward we consider only countries that have at least one news outlet (or political party) with a known political leaning, resulting in 1154905 (160200) posts from 19 (29) countries for the news (parties) dataset.

We define *C* as the number of distinct countries and *T* as the number of topics discussed. To mitigate potential biases due to the varying numbers of news outlets across countries, we construct a matrix *M* of dimensions *C* × *T*, where the entry *M*_*ij*_ represents the fraction of total engagement received in topic *j* by country *i*.

**Fig 4:**
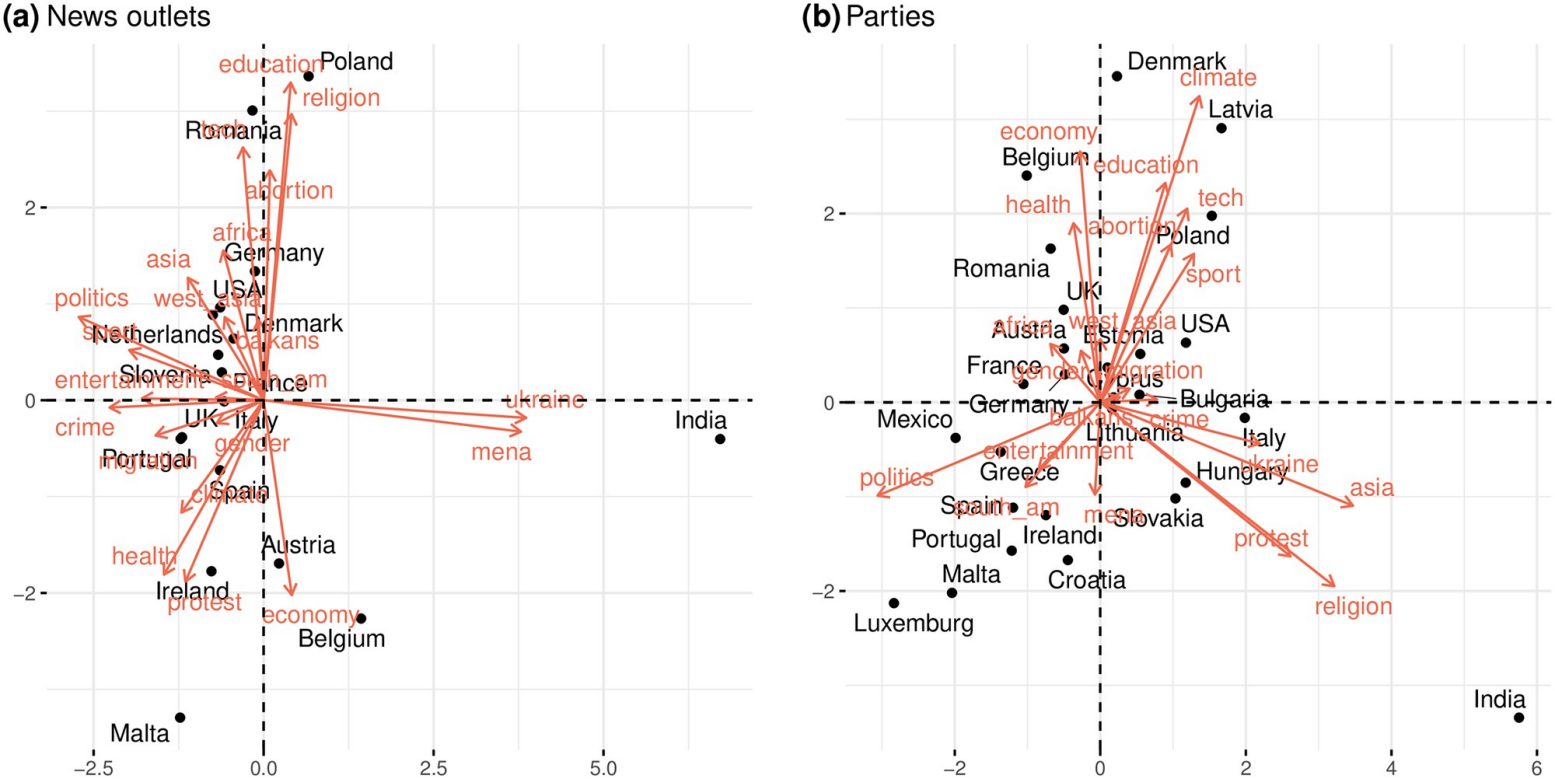
Biplot of most engaged topics for both news and parties datasets. Close points and vectors suggest similar profiles.

To distinguish between the discussions led by news outlets and political parties, we create two separate matrices, *M*^*news*^ and *M*^*parties*^. Principal Component Analysis (PCA) is then applied to these matrices to derive a two-dimensional representation that explains the most variance; further details can be found in the Materials and Methods section. Fig 4 presents the biplots from *M*^*news*^ and *M*^*parties*^.

As explained in Materials and Methods, the most important aspects that need to be noted in the figures are the magnitude of the vectors and the angle between each pair of vectors. The magnitude of the vector represents how well the variable is captured by the two dimensions, while the angle between two vectors is an expression of the correlation between them. In other words, two vectors of high magnitude that are roughly aligned indicate that the two corresponding variables are well correlated and clearly expressed by the dimensionality reduction processed. Conversely, vectors that are opposite (i.e., the cosine of their angle is −1) indicate that the two are anti-correlated. Notably, news outlets in India demonstrate unusually high engagement compared with other countries on topics such as the Middle East (abbreviated as MENA) and Ukraine. For most other countries, engagement patterns on the first principal component, which captures the largest variance, are relatively uniform, suggesting similar interest levels in entertainment, crime, and politics.

However, specific topics such as education, religion, technology, abortion, and africa show an inverse relationship with others like climate, health, protest, and economy. Countries like Ireland, Austria, Malta, and Spain are more engaged with the latter topics while less so with the former. From the perspective of political parties, engagement patterns vary significantly. For instance, parties in Northern European countries like Denmark, Belgium, and Latvia actively engage more with climate change, economy, education, health, abortion, technology, and sports. In contrast, topics such as protest, asia, religion, and ukraine obtain higher interactions in countries like Italy, India, Hungary, and Slovakia. This highlights a clear divergence in the focus of discussions between news outlets and political entities.

### Topics and political leaning

In the previous section, we extracted the most engaging topics from each country without considering the specific landscape of news outlets and political parties within those countries. In this section, we address this gap by applying a similar analysis distinctly for each nation, incorporating an additional dimension of political leaning (details are provided in the Materials and Methods section).

**Fig 5:**
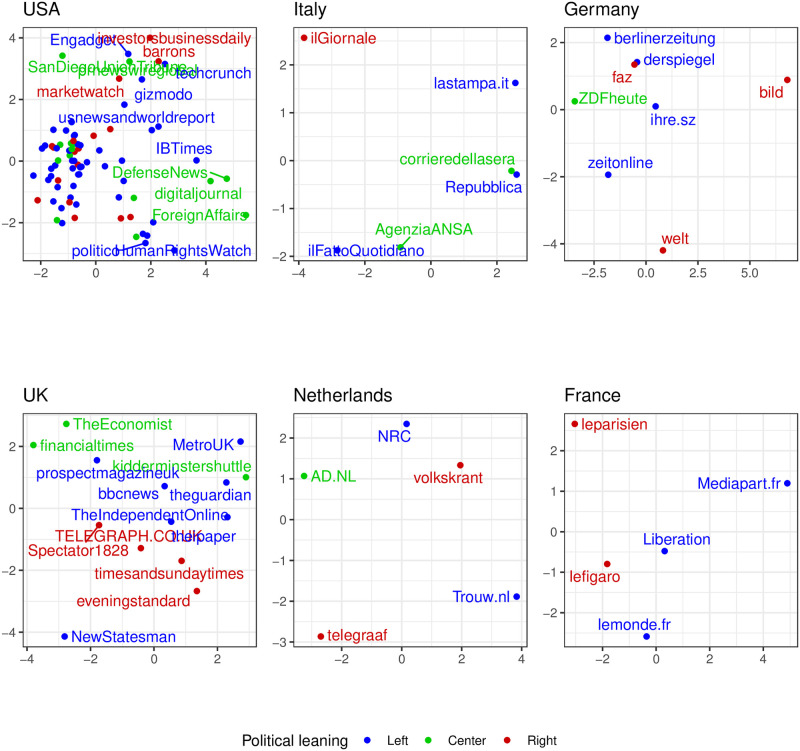
Biplot of news outlets grouped by engaged topics for countries with *n*_*c*_ ≥ 3. No clear clustering structure is present, suggesting that leaning does not play a role in which topics get more engagement.

We focus on the news outlets case (the parties are treated similarly). Following a method similar to the previous one, we consider a country *c* with *n*_*c*_ labeled news outlets discussing *m*_*c*_ topics. We construct the matrix *M*^*c*^ of dimension *n*_*c*_ × *m*_*c*_, where $M_{ij}^{c}$ represents the fraction of engagement received on topic *j* by news outlet *i*. Fig 5 shows the biplot resulting after applying PCA to *M*^*c*^ on each country having *n*_*c*_ ≥ 3.

We conduct a similar analysis for political parties, whose results are displayed in S6 Fig for all countries having *n*_*c*_ ≥ 3. By observing a stronger or weaker clustering structure, we can assess how discriminant the political leaning is in determining the engagement received by posts. In other words, if points of different political colors are overall well separated, this could suggest that the leaning plays an important role in the engagement received by different topics.

However, this is not true, as confirmed also by the silhouette values reported in S7 Fig. In the plot we can observe the mean Silhouette values of all the points in each biplot, considering the political leaning as the determinant of each cluster. Since almost all the scores have zero or negative values, we can conclude that political leaning does not determine good clusters. Therefore, it does not significantly influence how engagement is distributed across topics.

One possible explanation for the observed engagement patterns can be attributed to the overall narrative style of each page. Even when discussing the same topics, different pages may present perspectives that resonate more closely with specific audiences. However, due to the limitations of our classification approach, PCA primarily captures the general content themes without distinguishing between nuanced perspectives.

Despite this limitation and the potential imbalances in labeling across different countries, our findings suggest that news outlets and political parties with also different political leaning —left, center, or right— are likely to engage with similar topics.

As a further investigation, S8 Fig follows the same procedure used to obtain Fig 4, but showing the political affiliation of the ruling party of each country. To obtain this classification, we consider the leaning of the party of the head of government in each country, excluding the cases where there was a change in the ruling party under the period of observation or when there was a caretaker government (e.g., the Netherlands, Poland, or Portugal). We also excluded Slovakia, where the declared political affiliation of the ruling party is contested by the other parties of the same political family and is, therefore, controversial. With the same rationale, we conclude that the political leaning of the ruling party does not significantly affect engagement distributions for both news pages (*a*) and political parties (*b*). In fact, the Silhouette values are ≈ −0.22 for News and −0.14 for parties, suggesting no clear clustering structure. However, this result could be connected to the fact that most of our considered countries have high scores in democratic indices.

**Fig 6:**
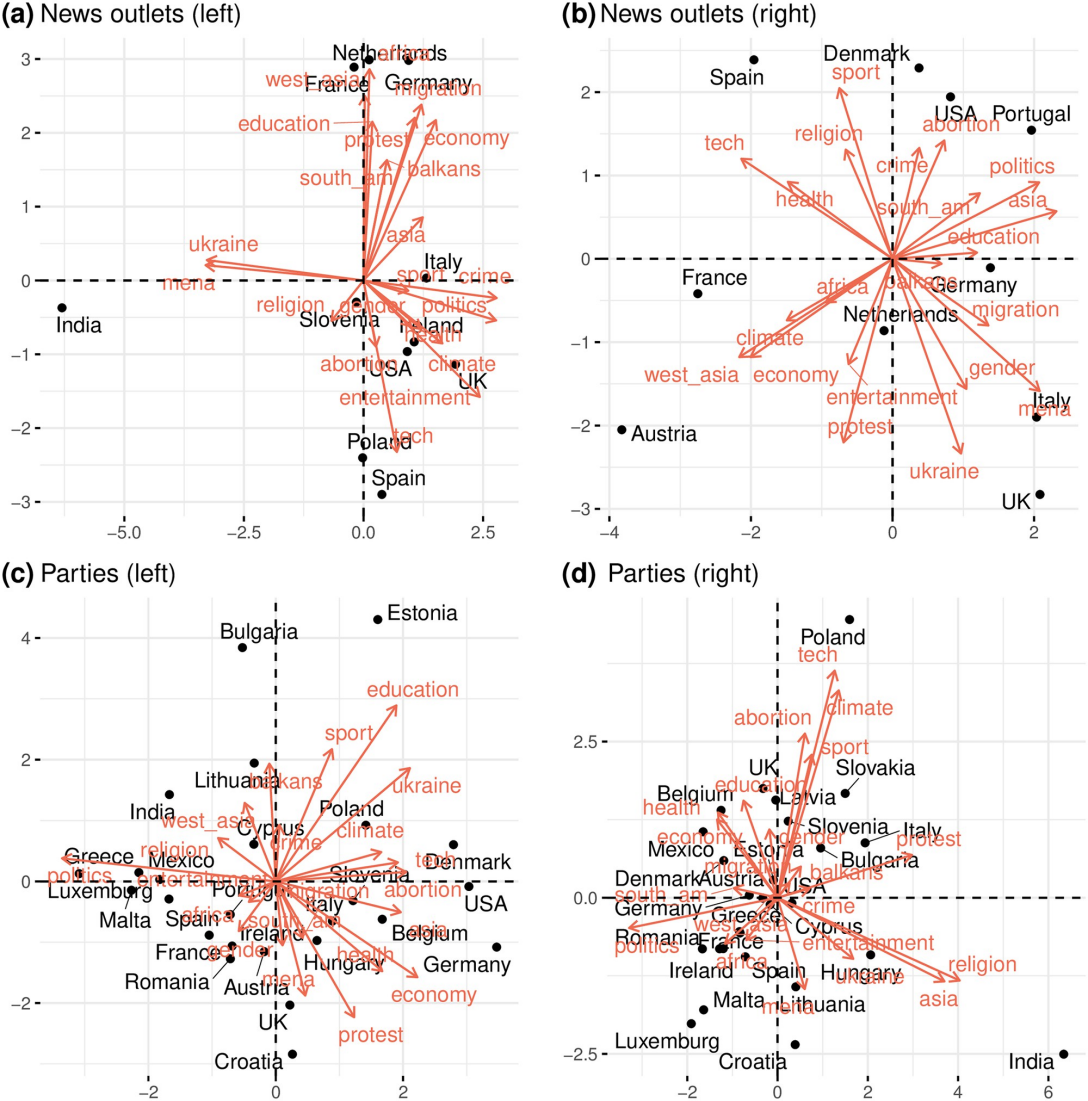
Most engaging topics of news outlets and parties if only a specific leaning is considered. Closeness between countries and topics suggests high engagement in that topic for that country.

To delve deeper into the engagement dynamics specific to each political leaning, we employ a similar analytical procedure. In particular, we construct a matrix *M*^*b*^ in which the rows are countries and columns are topics. Differently from before, the generic entry $M_{i,j}^{b}$ is the fraction of engagement received by news outlets of country *i*, having political leaning *b* ∈ {*left*, *right*}, in topic *j*. PCA is then applied to these matrices to yield a two-dimensional representation of the topic discussed by a specific political leaning in each country. The results are reported in Fig 6.

We observe that left news outlets in Poland, Spain, USA, Ireland and UK are getting high engagement in topics such as abortion, religion, climate and tech. At the same time Netherlands, France and Germany are more focused on education, economy, africa and migration. On the other hand, right news outlet in Italy, Germany, UK and Netherlands are getting high engagement in topics such as gender, migration and ukraine. Interestingly, USA, Portugal, Denmark and Spain are more interested in religion, abortion, sport and crime. Finally, right news outlets from France and Austria seem to get a higher engagement with the climate topic than other countries.

Fig 6(*c*) and 6(*d*) depict the results of the same analysis for Political Parties. In this case we observe even more differences between each country, suggesting that political parties from different countries, even if with the same political leaning—left or right—tend to engage in varying and different topics.

Finally, we aim to obtain a representation of the topics discussed by each political leaning in each country. To do that, we employ the following procedure: For each country *c*, we construct a matrix *M*^*c*^ of dimension 3 × *m*_*c*_ where the rows represent the political leanings of the news outlets—*left, center*, and *right*. The entry $M_{ij}^{c}$ denotes the fraction of engagement received by news outlets with political leaning *i* in topic *j*. PCA is then applied to obtain a low-dimensional representation capturing the most significant aspects of the data.

**Fig 7:**
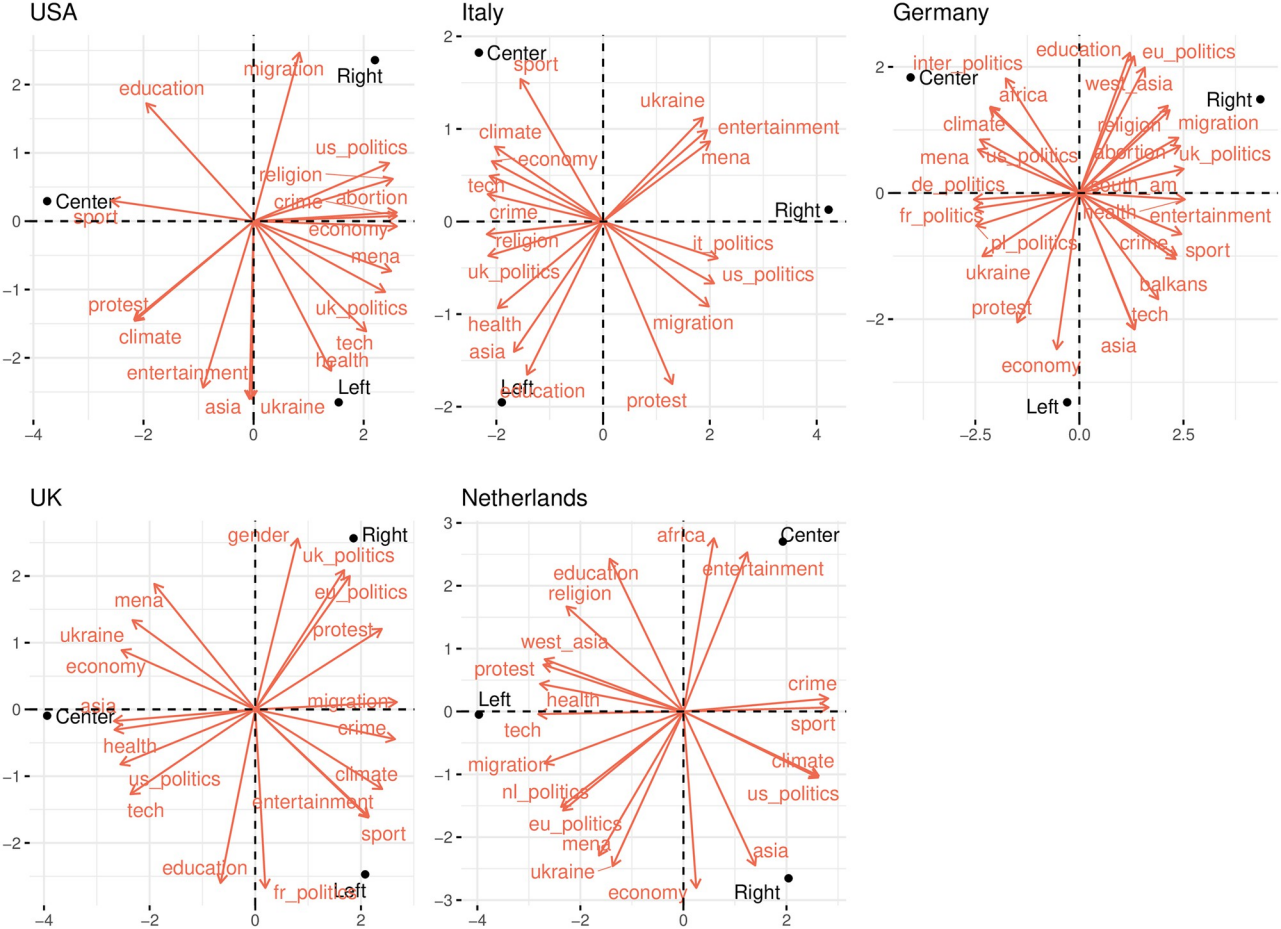
Most engaging topics of news outlets by political leaning. Closeness between a political leaning and a topic indicate high engagement in that topic for that political leaning.

The resulting biplots presented in Fig 7 generally indicate that right-leaning news outlets attract more engagement on topics related to politics, religion, and migration, whereas left-leaning news outlets are more actively engaged in topics like education, health, and technology. Additionally, the angles between topics suggest a slight negative correlation between the topics favored by the left and right, indicating a moderate level of, overall, topic segregation.

**Fig 8:**
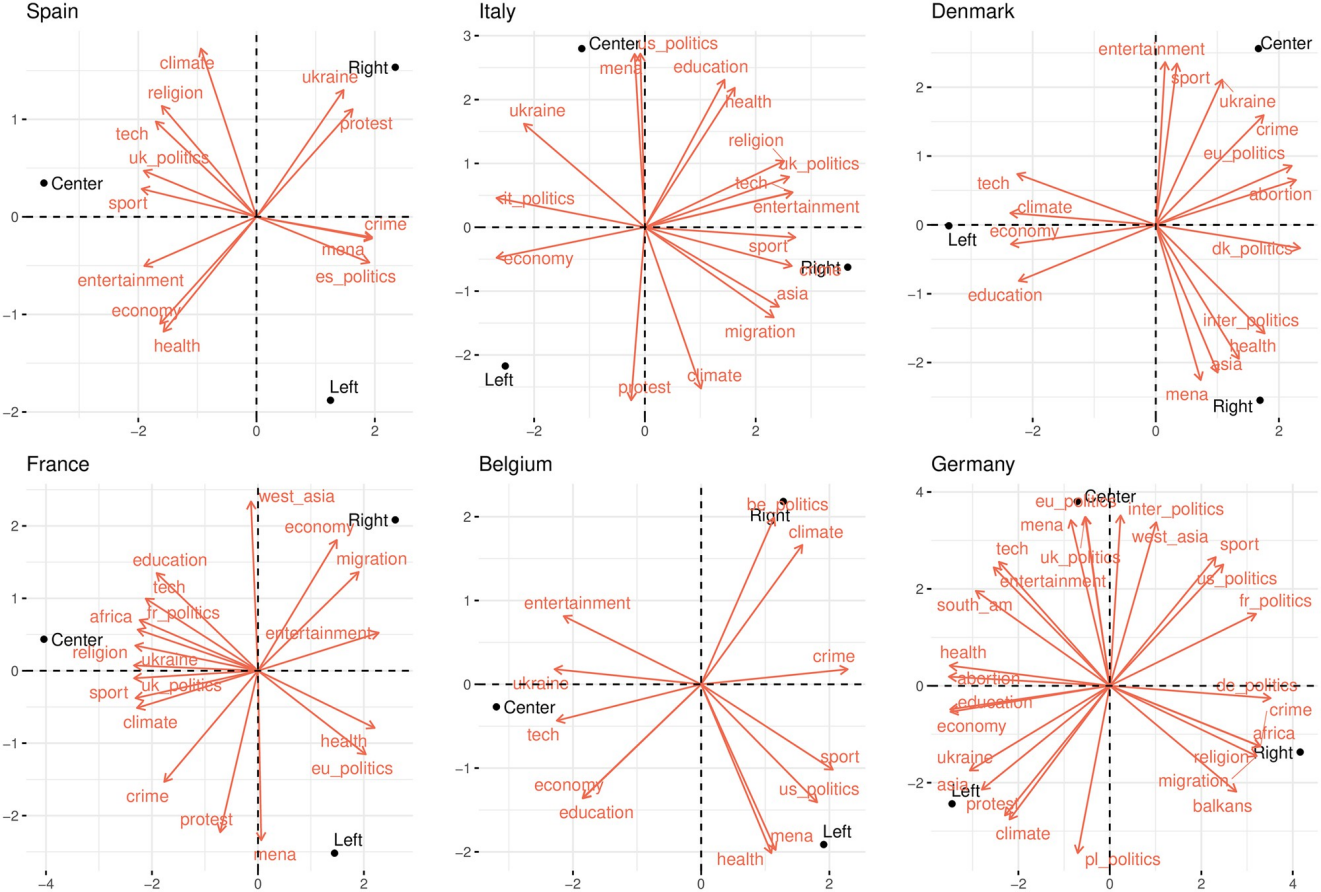
Most engaging topics of parties by political leaning. Closeness between a political leaning and a topic indicate high engagement in that topic for that political leaning.

Similarly, Fig 8 displays the engagement patterns by political leaning for a subset of countries in the parties dataset. Despite some exceptions, we see similar trends to the ones observed in the News dataset. The complete results are detailed in S9 Fig.

## Limitations

We acknowledge that our work presents some limitations, which we discuss in this section. Firstly, our dataset contains only aggregate Facebook data. This is due to project manageability, as well as the fact that most, if not every, political party and news agency has a Facebook page. Moreover, pages of news agencies and political parties are likely to post the same content on multiple networks, so the analysis of the posted content is not likely to significantly differ. Moreover, due to the absence of users’ longitudinal data, it is impossible to know the effects of a certain kind of posts on users, including the degree of uncivil behavior or the exact selectivity of their interactions. Lastly, we rely on external classification for the leanings of news agencies, which is ultimately decided by humans and concentrated on rich, western countries. To overcome these limitations, future works might consider a multiplatform dataset containing users’ longitudinal data.

Despite these limitations, we believe that our work still provides an important insight into the shape of the media landscape during multiple election campaigns.

## Conclusion

This study examines how political leanings and media outlets engage audiences on social media during critical events such as global elections.

While both news outlets and political parties often address similar broad topics, we observe differences in the intensity and nature of engagement based on political orientation. Right-leaning outlets and parties tend to prioritize politics, religion, and migration, whereas left-leaning groups focus more on education, health, and technology. However, our results also suggest that the divide is not absolute. Even polarized groups can find common ground on specific issues, though they approach them from unique perspectives.

These findings hold valuable insights for policymakers, media strategists, and researchers aiming to create messages that bridge divides and enhance public discourse. Understanding these engagement patterns can inform the design of social media campaigns that account for ideological differences and audience preferences. Additionally, the clustering of users around specific political pages, combined with exposure to limited topics, underscores the risk of polarization and even radicalization in online environments. Future works would consider a broader dataset that tracks user activity and measures the toxicity of interactions to enhance our understanding of these dynamics.

Finally, we believe that an evidence-based understanding of the debate at the national level on universally important topics, such as climate change, global conflicts, and public health issues, can offer valuable insights into public opinion, help identify areas of consensus and disagreement and help develop evidence-based policies that are more likely to be supported and implemented.

## Supporting information

S1 Fig Number of weekly posts from the four most popular topics discussed by American news organizations (a) and politicians (b). (TIF)

S2 Fig Average number of likes per week received by posts from the four most popular topics discussed by American news organizations (a) and politicians (b). (TIF)

S3 Fig Number of posts in each cluster of both topic modelling processes. (TIF)

S4 Fig Reaction distributions to news posts across countries. (TIF)

S5 Fig Reaction distributions to posts from political parties across countries. (TIF)

S6 Fig Biplot of parties grouped by engaged topics. (TIF)

S7 Fig Siluette plot of news (top) and political parties (bottom). (TIF)

S8 Fig Biplot of posts from news outlets and political parties from each country. (TIF)

S9 Fig More engaging topics for parties. (TIF)

S1 Table List of pages used in the study. (PDF)

S2 Table A sample of posts assigned to the first cluster by the LDA model and the BERTopic-based topic modeling. (PDF)
