# Supplementary figures and images for: Mapping the global election landscape on social media in 2024
Source: PLoS One. 2025 Feb 5;20(2):e0316271. doi: 10.1371/journal.pone.0316271 (PMC11798462; doi:10.1371/journal.pone.0316271)

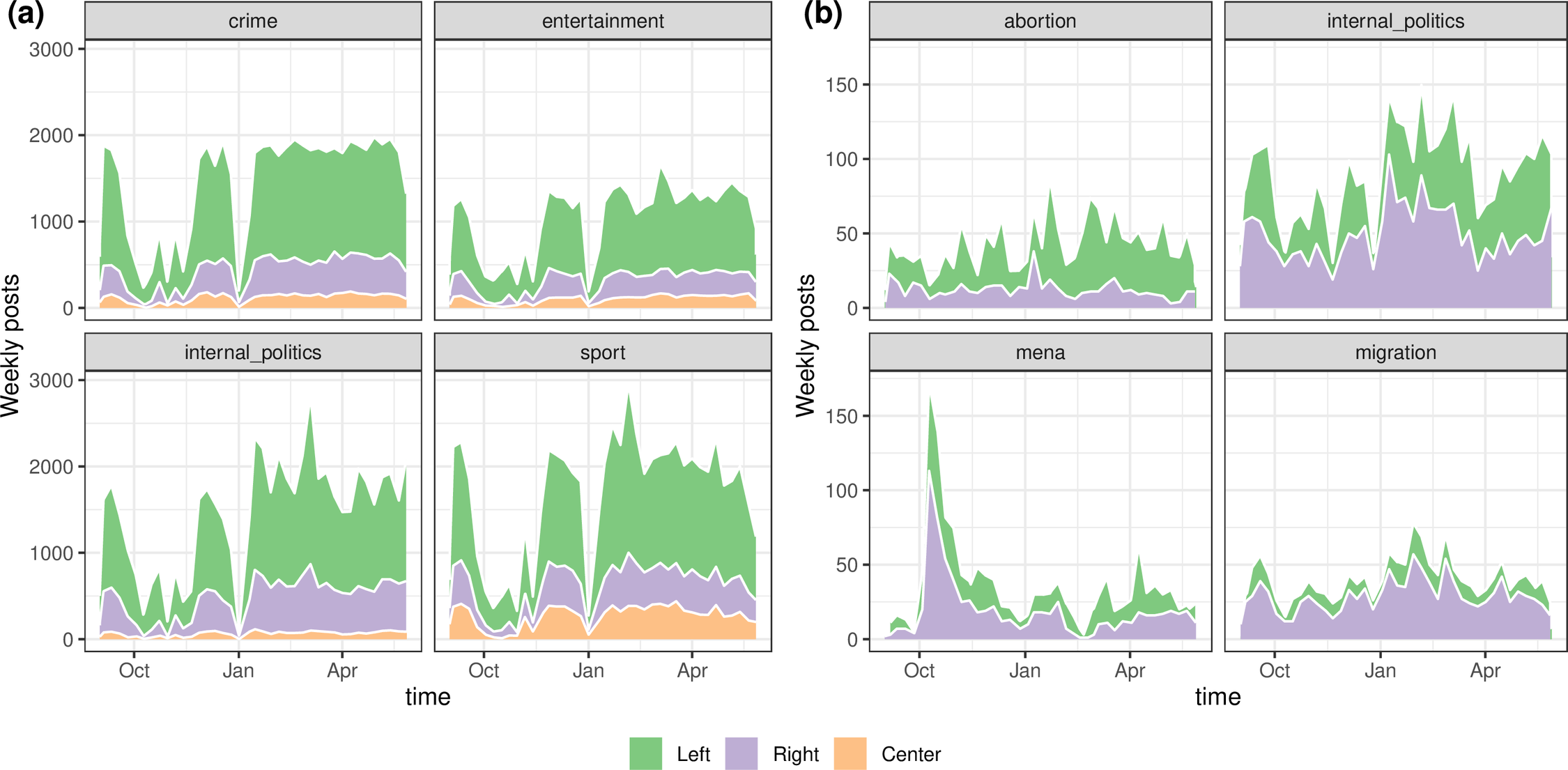

Supplement: S1 Fig — (TIF) [file pone.0316271.s001.tif]

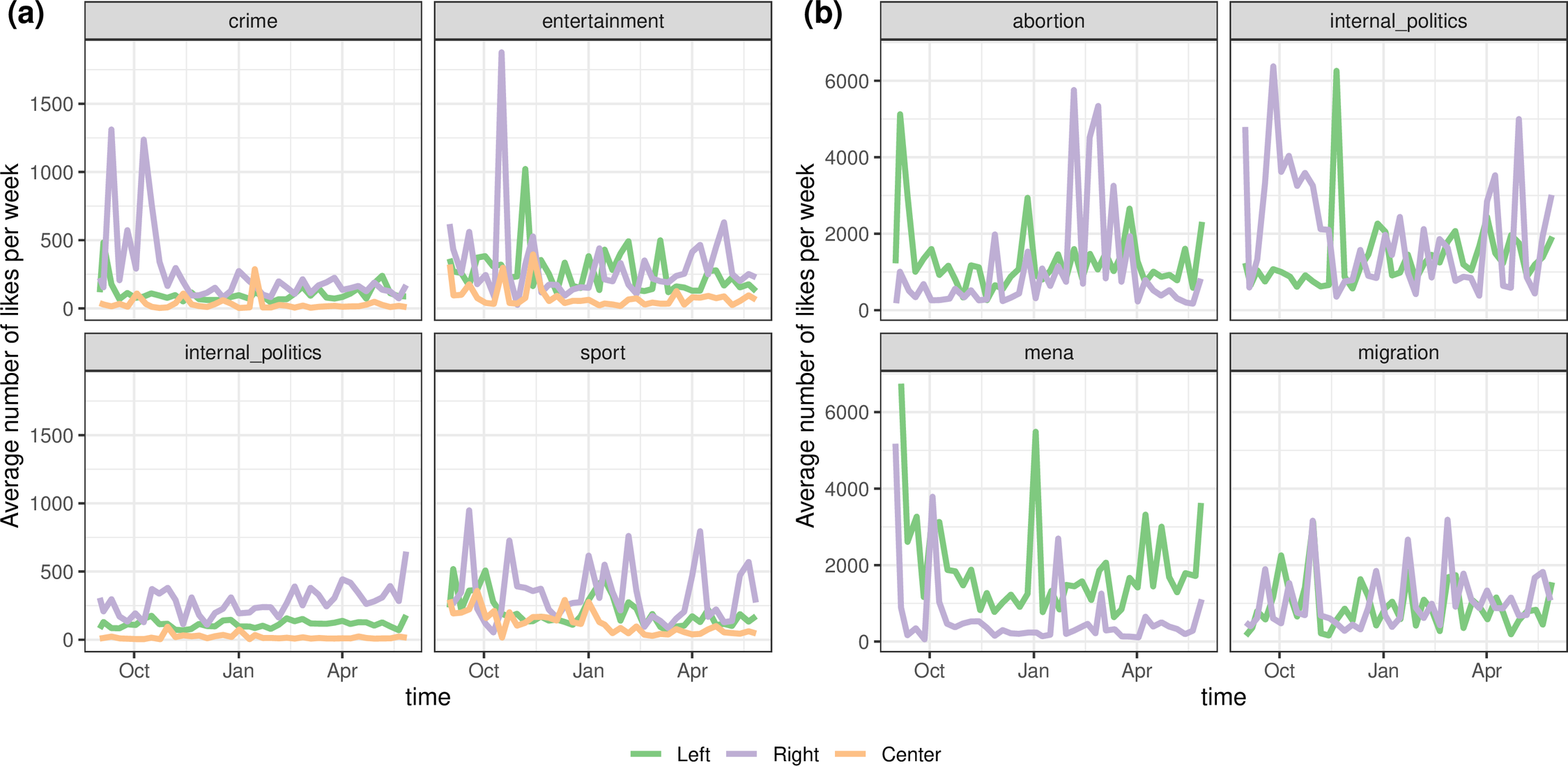

Supplement: S2 Fig — (TIF) [file pone.0316271.s002.tif]

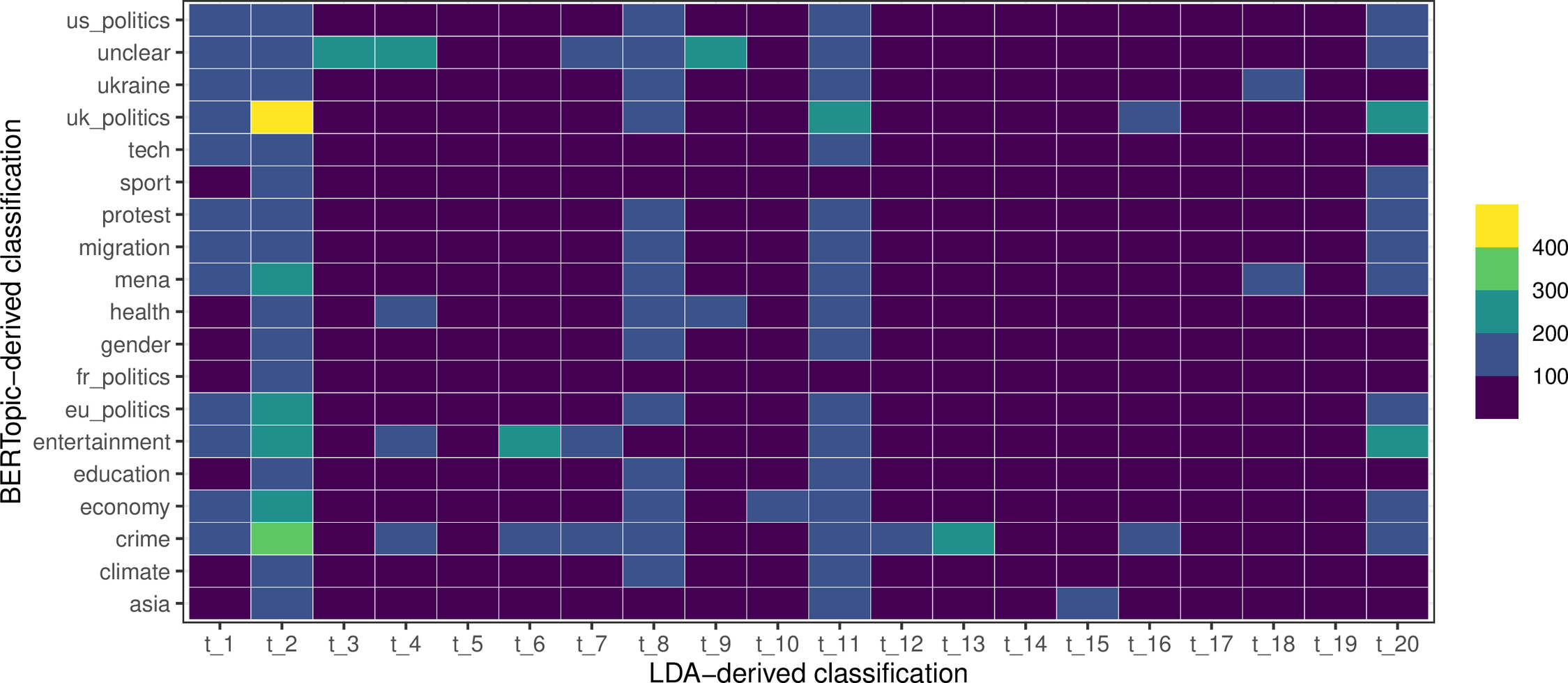

Supplement: S3 Fig — (TIF) [file pone.0316271.s003.tif]

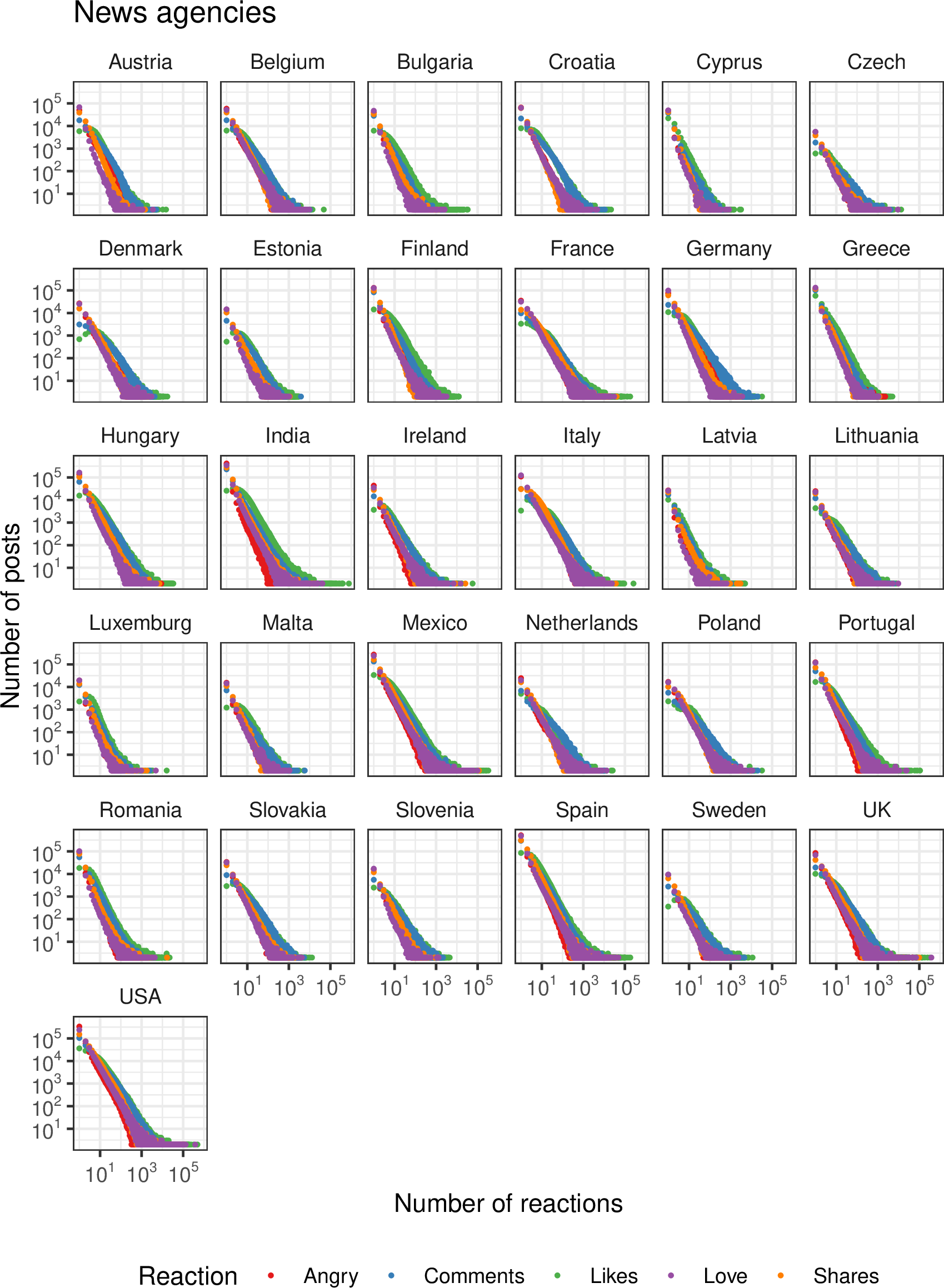

Supplement: S4 Fig — (TIF) [file pone.0316271.s004.tif]

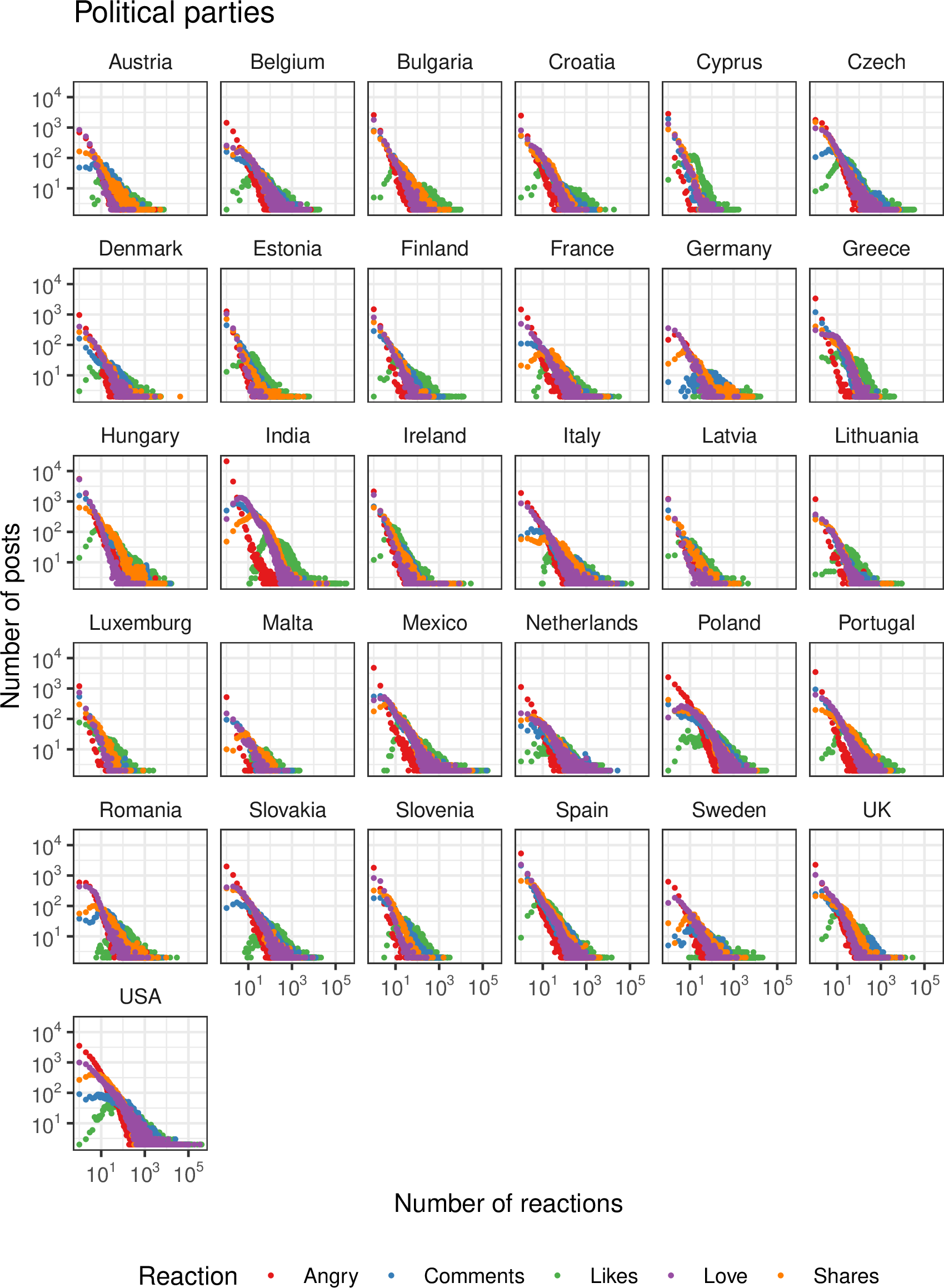

Supplement: S5 Fig — (TIF) [file pone.0316271.s005.tif]

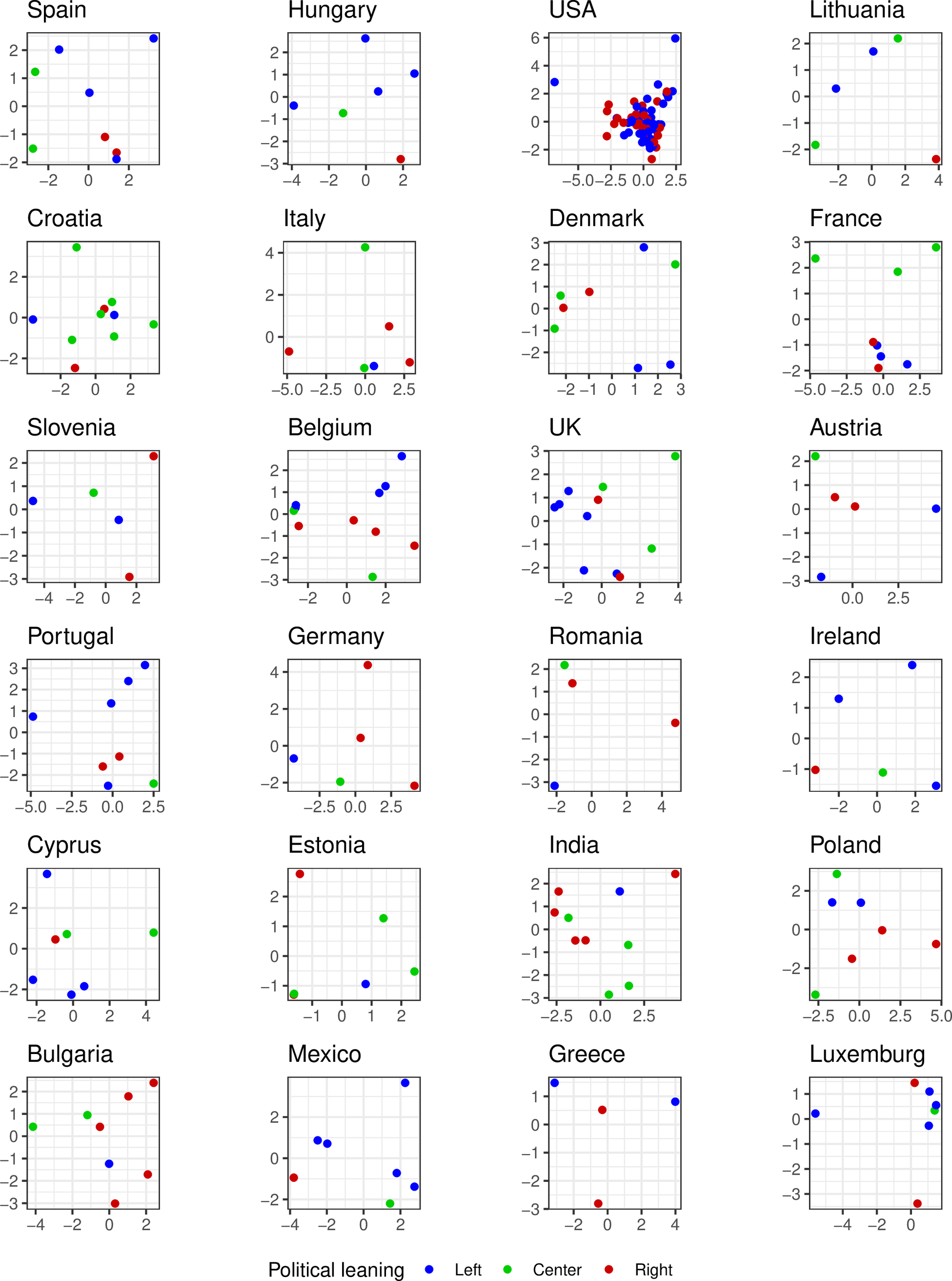

Supplement: S6 Fig — (TIF) [file pone.0316271.s006.tif]

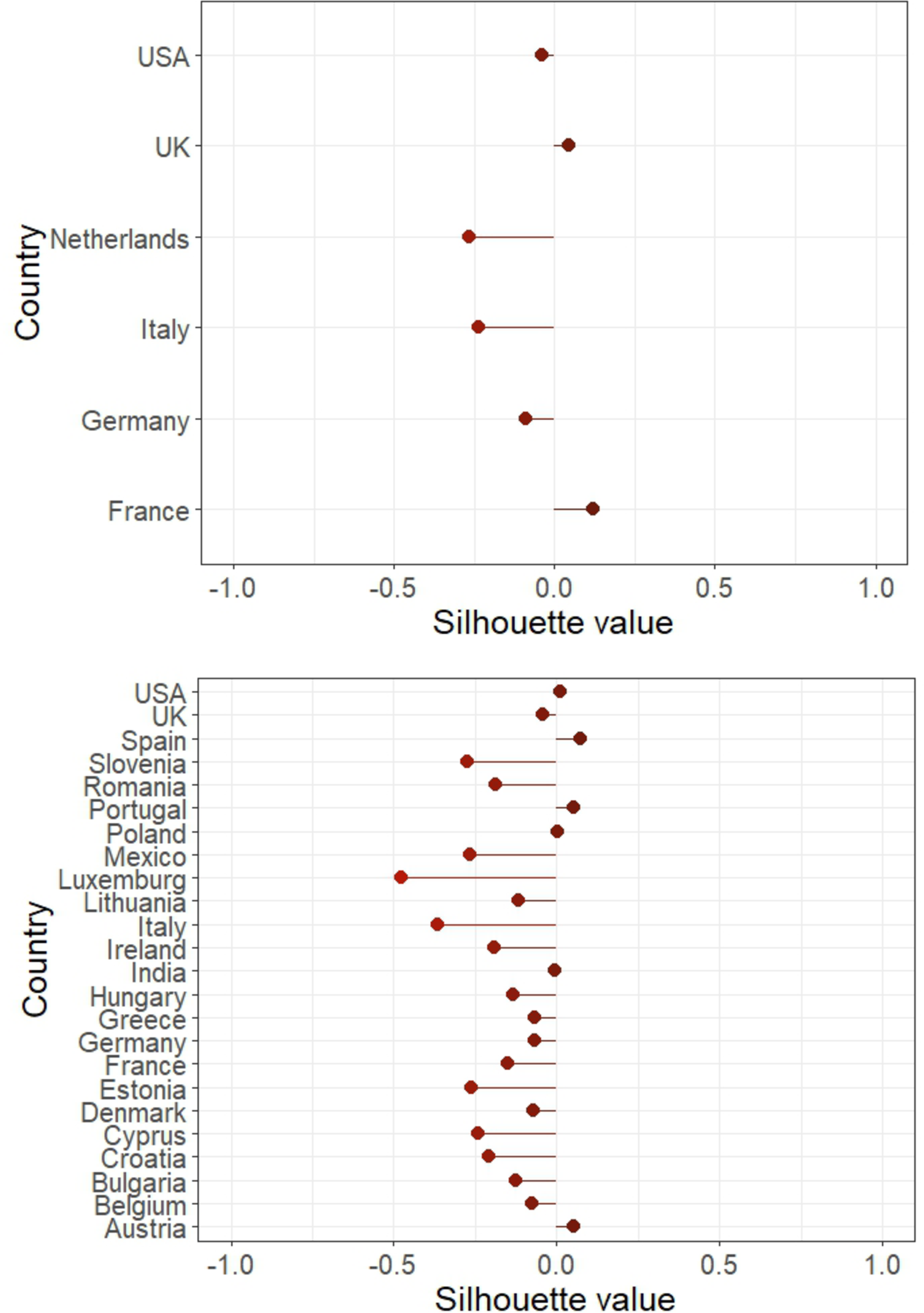

Supplement: S7 Fig — (TIF) [file pone.0316271.s007.tif]

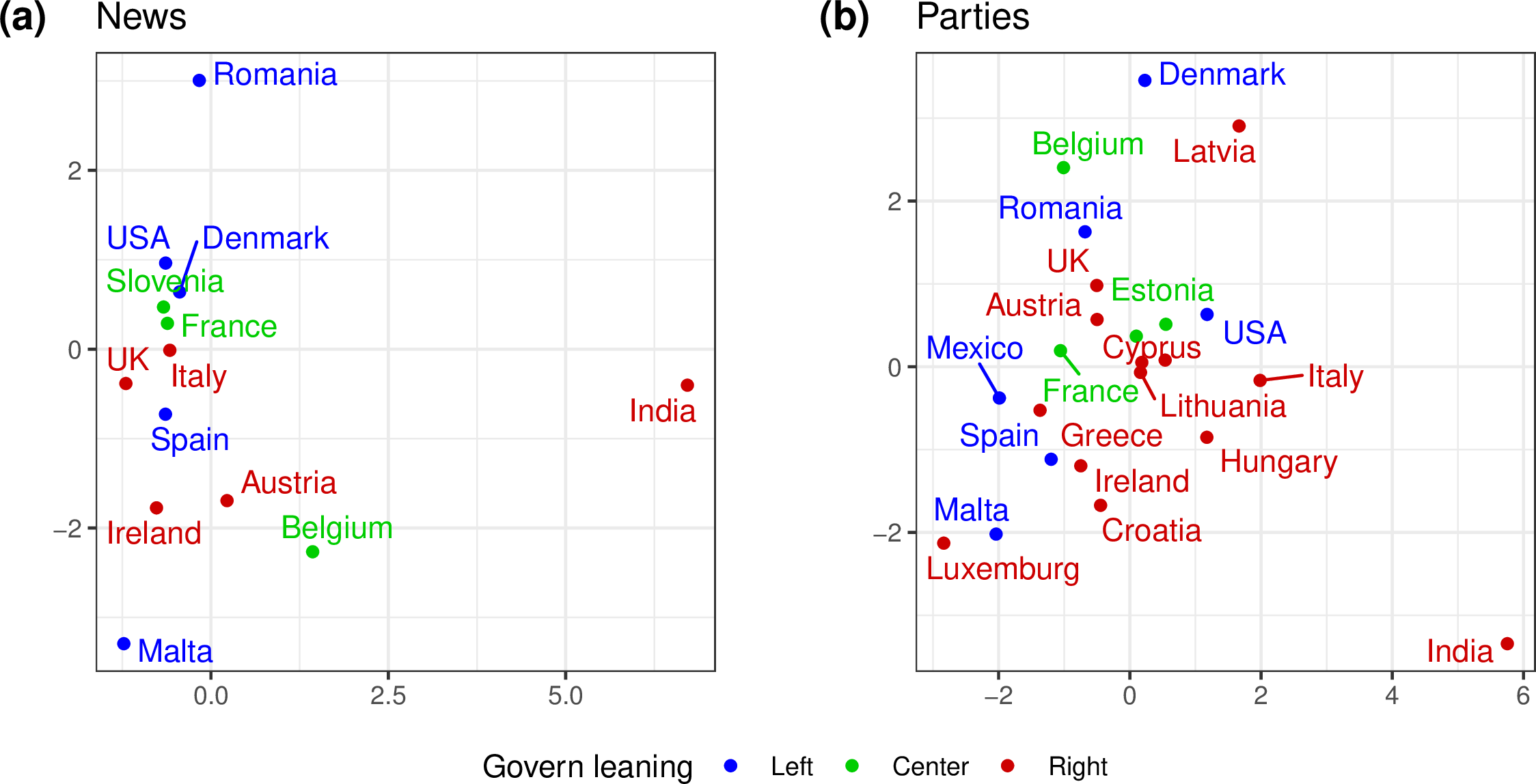

Supplement: S8 Fig — (TIF) [file pone.0316271.s008.tif]

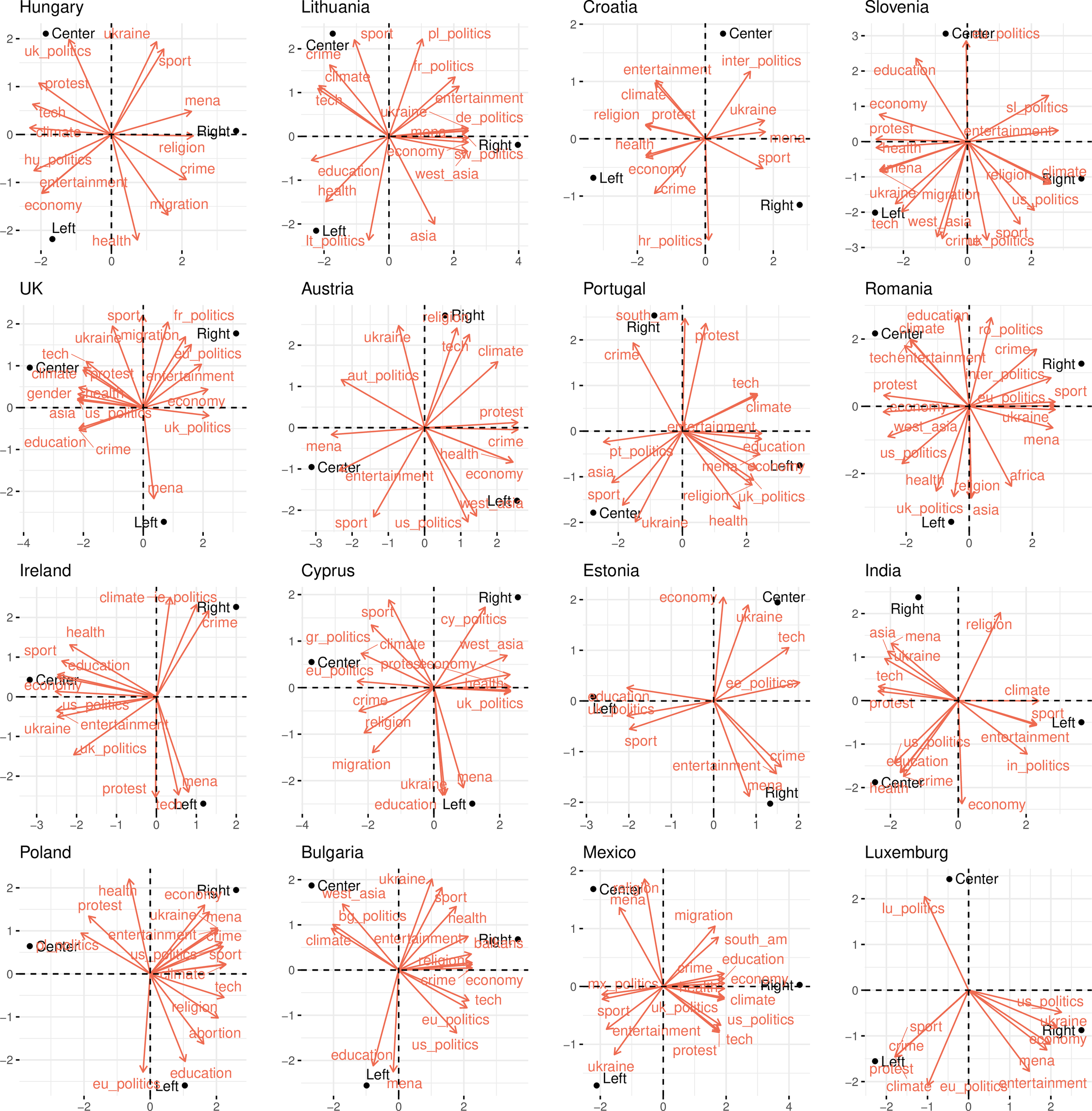

Supplement: S9 Fig — (TIF) [file pone.0316271.s009.tif]
