# Supplementary material for: Mapping the global election landscape on social media in 2024
Source: PLoS One. 2025 Feb 5;20(2):e0316271. doi: 10.1371/journal.pone.0316271 (PMC11798462; doi:10.1371/journal.pone.0316271)
Supplement: S1 Table — (PDF) [file pone.0316271.s010.pdf]

Supporting information

Table 1. List of pages used in the study.

user_name	facebook_id	country	type
volksblatt.at	100045120062884	Austria	news
neuetageszeitung	100063825434906	Austria	news
derStandardat	100064428591639	Austria	news
oe24.at	100064437983860	Austria	news
TirolerTageszeitung	100064529960707	Austria	news
DiePressecom	100064626826428	Austria	news
heute	100064758518481	Austria	news
salzburger.nachrichten	100064915682949	Austria	news
kleinezeitung	100064954141289	Austria	news
KURIER	100071245366349	Austria	news
degentenaar	100048634435435	Belgium	news
detijd	100063616747864	Belgium	news
metrobelgique	100063718684113	Belgium	news
GRENZECHO.net	100064316070895	Belgium	news
hetbelangvanlimburg	100064480522666	Belgium	news
demorgen	100064499871779	Belgium	news
lesoirbe	100064530170861	Belgium	news
nieuwsblad.be	100064557829768	Belgium	news
lavenir.net	100064593223358	Belgium	news
lalibre.be	100064623613822	Belgium	news
destandaard	100064703858214	Belgium	news
BrusselsTimes	100064717417376	Belgium	news
Gazetvanantwerpen	100064773730560	Belgium	news
lechobe	100064868255048	Belgium	news
telegraph.bg	100063585639566	Bulgaria	news
borbavt	100063679266749	Bulgaria	news
trud.bg	100063938644879	Bulgaria	news
24chasa	100064254836629	Bulgaria	news
sega.bulgaria	100064521319288	Bulgaria	news
vestnikbanker	100064528636494	Bulgaria	news
standartnews	100064653135465	Bulgaria	news
dnevnik	100064734488972	Bulgaria	news
dnesbulgaria	100083147509857	Bulgaria	news
montpress	100048912222554	Bulgaria	news
Glasistre.hr	100028989566235	Croatia	news
Glas.Slavonije	100056653586924	Croatia	news
novilist	100063474323974	Croatia	news
zadarski.hr	100063678693713	Croatia	news
Dubrovackivjesnik	100063707313492	Croatia	news
poslovni.hr	100064380113366	Croatia	news
vecernji	100064521411238	Croatia	news
slobodnadalmacija	100064622562165	Croatia	news
jutarnji.list	100064691530707	Croatia	news
Haravgi	100050317346041	Cyprus	news
simerini.news	100063526965440	Cyprus	news
politisnewspaper	100063639114710	Cyprus	news

AlithiaMedia	100063679384921	Cyprus	news
CyprusMail	100063685807707	Cyprus	news
phileleftheros	100064363402618	Cyprus	news
cynewseu	100064818153196	Cyprus	news
denik.cz	100063539801434	Czech	news
denikpravo	100063543933911	Czech	news
Blesk.cz	100064364576701	Czech	news
hospodarky	100064371956072	Czech	news
MladaFrontaDNES	100063855720670	Czech	news
lidovenoviny	100057511403915	Czech	news
copenhagenpost	100052648913632	Denmark	news
arbejderen	100057062757080	Denmark	news
Weekendavisen	100063478336171	Denmark	news
kristeligt	100063617634377	Denmark	news
berlingske	100064331127072	Denmark	news
ekstrabladet	100064630966309	Denmark	news
jyllandsposten	100064681511805	Denmark	news
borsen.dk	100064732057174	Denmark	news
politiken	100064763048362	Denmark	news
ditbt	100064835255015	Denmark	news
dagbladetinformation	100064985370038	Denmark	news
saartehaal	100060830390957	Estonia	news
aripaev	100063516791933	Estonia	news
eestipaevaleht	100063559421999	Estonia	news
postimees	100064361962471	Estonia	news
Ohtuleht	100064573758796	Estonia	news
helsinginsanomat	100064708989284	Finland	news
Hufvudstadsbladet	100063691744768	Finland	news
esaimaa	100063675423295	Finland	news
lansisuomi	100063279017323	Finland	news
ksmlfi	100046602140170	Finland	news
KalevaOy	100064052225054	Finland	news
vasabladet.fi	100063762784502	Finland	news
HameenSanomat	100064209157034	Finland	news
savonsanomat1	100064139465052	Finland	news
kainuunsanomat.fi	100034431856916	Finland	news
	100043574843659	Finland	news
lansiuusimaa	100043967245661	Finland	news
satakunnankansa	100029619590706	Finland	news
aamulehti	100058005080883	Finland	news
salonseudunsanomat	100063611108579	Finland	news
Ilkkapohjalainen.fi	100038120417469	Finland	news
lapinkansa	100063761552409	Finland	news
uusimaa.fi	100063707643218	Finland	news
iisalmensanomat	100064143879954	Finland	news
kauppalehti.fi	100063543011493	Finland	news
abounderrattelser	100063771992702	Finland	news
taloussanomat	100063640656805	Finland	news
kouvolansanomat	100063610268661	Finland	news
nyaaland	100041521376428	Finland	news

lansisavo	100049970485384	Finland	news
turunsanomat	100063645114913	Finland	news
kymensanomat	100063593829272	Finland	news
SanomalehtiKarjalainen	100023966306947	Finland	news
Keskilaakso	100063753334909	Finland	news
osterbottenstidning	100063702543765	Finland	news
keskipohjanmaa	100063631387875	Finland	news
kansanuutiset	100041901475678	Finland	news
aamuposti	100063626048399	Finland	news
demokraatti.fi	100063032390600	Finland	news
alandstidningen	100063661385369	Finland	news
itahame	100028107566237	Finland	news
itasavo	100041220175772	Finland	news
vastranyland	100040087584524	Finland	news
tyrvaansanomat	100053561535865	Finland	news
KarjalanH	100063548770229	Finland	news
lequipe.fr	100044348443220	France	news
Contrepoints	100063727893534	France	news
lacroix.journal	100063778574382	France	news
lemonde.fr	100064539140308	France	news
lefigaro	100064714449291	France	news
lesechos	100064763164049	France	news
humanite.fr	100064860845478	France	news
Mediapart.fr	100064909983213	France	news
20minutes	100064974150667	France	news
Liberation	100064986330109	France	news
leparisien	100069002424655	France	news
latribune	100078075303669	France	news
junge.welt	100046632146318	Germany	news
welt	100053495553917	Germany	news
wznewsline	100060895134337	Germany	news
FrankfurterRundschau	100063650589063	Germany	news
nd.aktuell	100063791130047	Germany	news
rponline	100064293565184	Germany	news
berlinerzeitung	100064302865032	Germany	news
taz.kommune	100064324914179	Germany	news
bild	100064507341597	Germany	news
Tagesspiegel	100064522520895	Germany	news
faz	100064567848864	Germany	news
handelsblatt	100064653795335	Germany	news
derspiegel	100064772348380	Germany	news
ZDFheute	100064809428075	Germany	news
zeitonline	100064829557159	Germany	news
ihre.sz	100064830484979	Germany	news
stern	100064869632404	Germany	news
DromosThsAristeras	100062329604090	Greece	news
tomanifesto.gr	100063536504637	Greece	news
AvgiOnline	100063621801820	Greece	news
eleftheriora	100063696652610	Greece	news
vradini.gr	100063734023729	Greece	news

karfitsa.gr	100063749525212	Greece	news
dimokratianews.gr	100063791012388	Greece	news
paron.gr	100063872528301	Greece	news
EleftherosTypos	100064014158824	Greece	news
newsmakedonias	100064023874001	Greece	news
efmpam	100064095483038	Greece	news
pringr1	100064107127357	Greece	news
paraskhnio	100064119580269	Greece	news
taneagr	100064440201946	Greece	news
www.parapolitika.gr	100064496034215	Greece	news
efimerida.syntakton	100064539228165	Greece	news
topontiki	100064968360805	Greece	news
valaszonline	100032983166134	Hungary	news
UngarnHeute	100032983640728	Hungary	news
dailynewshungary	100039554211314	Hungary	news
vasarnap.hu	100043995405628	Hungary	news
magyarkurir	100047641015458	Hungary	news
Metropol.Napilap	100052786192503	Hungary	news
telexhu	100054073007151	Hungary	news
media1hu	100063327212183	Hungary	news
BudapesterZeitung	100063567550956	Hungary	news
magyarnemzet.hu	100063619687797	Hungary	news
nepszavaonline	100063643492521	Hungary	news
magyarjelen	100063746605581	Hungary	news
pestibulvar	100063773312676	Hungary	news
velvethu	100063912728962	Hungary	news
ezalenyeg.hu	100064082855223	Hungary	news
magyarinfo	100064324014009	Hungary	news
24ponthu	100064331216521	Hungary	news
Borsonline.Bors.Szorakoztato.Napilap	100064364726814	Hungary	news
AlfahirHirportal	100064516427295	Hungary	news
economx.hu	100064544927214	Hungary	news
Indexhu	100064585093978	Hungary	news
portfolio.hu	100064669694898	Hungary	news
168ora	100064672989973	Hungary	news
hungarytoday	100064734217203	Hungary	news
blikkhu	100064763644053	Hungary	news
444.hu	100064881090307	Hungary	news
atlatszo.hu	100067717692359	Hungary	news
AboutHungary	100069124123415	Hungary	news
OrigoHirek	100069355143217	Hungary	news
magyarhirlapfb	61550744906230	Hungary	news
kitekinto	100034708220685	Hungary	news
journalfrancophonedebudapest	100036175584070	Hungary	news
dailythanthi	100064236057276	India	news
mathrubhumidotcom	100064275505737	India	news
Madhyamam	100064321164121	India	news
patrikahindinews	100064489187935	India	news
hindustantimes	100064660902473	India	news
AnandabazarSocial	100064721134943	India	news

Sakshinews	100064739974516	India	news
manoramaonline	100064748133924	India	news
Pkesarionline	100064770070599	India	news
enavabharat	100064778105936	India	news
TheNavbharatlive	100064778105936	India	news
DailyGreaterKashmir	100064778583465	India	news
DeepikaNewspaper	100064883342294	India	news
sirajonlive	100064934820717	India	news
sangbadpratidin.in	100066353026637	India	news
thehindu	100069641816229	India	news
eenaduonline	100070918585406	India	news
DinamalarDistrictNews	100076824197305	India	news
lokmat	100077795915005	India	news
IrishHerald	100063540608271	Ireland	news
IrishDailyStar	100063793292361	Ireland	news
theirishsun	100064242387150	Ireland	news
irishmirror	100064380833692	Ireland	news
irishtimes	100064447823624	Ireland	news
IrishExaminer	100064837142381	Ireland	news
Independent.ie	100064865345158	Ireland	news
domanigiornale	100039815302405	Italy	news
tgla7	100046840735192	Italy	news
ilFattoQuotidiano	100053429766843	Italy	news
Ildubbionews	100063545110411	Italy	news
milanofinanza	100063625867727	Italy	news
ilsole24ore	100064360407072	Italy	news
corrieredellasera	100064368746696	Italy	news
SkyTG24	100064554647327	Italy	news
liberonews	100064555695145	Italy	news
AgenziaANSA	100064615457043	Italy	news
fanpage.it	100064653281114	Italy	news
ilmanifesto	100064667114396	Italy	news
quotidianolaverita	100064678177949	Italy	news
tgcom24	100064680279179	Italy	news
avvenire.it	100064688078819	Italy	news
RaiNews	100064695876217	Italy	news
ilfoglio	100064802288107	Italy	news
ilGiornale	100064829017156	Italy	news
Repubblica	100070283104562	Italy	news
lastampa.it	100071317846404	Italy	news
DienaLV	100046659913828	Latvia	news
DienasBizness	100057396762122	Latvia	news
wwwIRlv	100063579130070	Latvia	news
LatvijasAvize	100063777901867	Latvia	news
nralv	100064124948564	Latvia	news
LatvijasVestnesis	100064738241163	Latvia	news
verslozinios	100063528673426	Lithuania	news
respublika.lt	100063770723844	Lithuania	news
lrytaslt	100064693330778	Lithuania	news
TheLithuaniaTribune	100060215265123	Lithuania	news

telecran.luxemburg	100044189815158	Luxemburg	news
Tageblatt.lu	100046508845546	Luxemburg	news
woxx.lu	100062610230174	Luxemburg	news
revue.lu	100063003290239	Luxemburg	news
	100063581950772	Luxemburg	news
wort.lu.de	100063889300522	Luxemburg	news
letzjournal	100063949338917	Luxemburg	news
LeQuotidien.lu	100063965316913	Luxemburg	news
brozlv	100065158352515	Luxemburg	news
lessentielonline	100067298233955	Luxemburg	news
goosch.lu	100063951463477	Luxemburg	news
IllumMediaToday	100031468608077	Malta	news
TheMaltaIndependent	100064088889987	Malta	news
NetNewsMalta	100064732508964	Malta	news
ONE.com.mt	100064779873833	Malta	news
timesofmalta	100064984770069	Malta	news
Cuartopodermx	100058908474479	Mexico	news
cambiodemichoacan	100063463493908	Mexico	news
periodicoelautonomo	100063920115779	Mexico	news
	100063934808025	Mexico	news
CampecheHOYmx	100064069412021	Mexico	news
periodicoeldebate	100064293504836	Mexico	news
elimparcialcom	100064370546242	Mexico	news
MilenioDiario	100064424424683	Mexico	news
criteriohidalgo	100064456642032	Mexico	news
ElDiarioDelYaqui	100064459191590	Mexico	news
lajornadaonline	100064499151846	Mexico	news
ElUniversalOnline	100064562299062	Mexico	news
LaCronicaDeHoy	100064643085866	Mexico	news
abcnoticiasmty	100064728754683	Mexico	news
revistaproceso	100064917032757	Mexico	news
ExcelsiorMex	100064918140477	Mexico	news
ElEconomista.mx	100064918230835	Mexico	news
elinformador	100067238112404	Mexico	news
DiariodeYucatan	100070081782559	Mexico	news
elheraldodemexico	100071338323935	Mexico	news
eldebatecom	100076403791728	Mexico	news
	100083883133633	Mexico	news
	100031928451274	Mexico	news
Trouw.nl	100063462223346	Netherlands	news
refdag	100063773238966	Netherlands	news
het.financieele.dagblad	100063973664746	Netherlands	news
nederlandsdagblad	100064118551566	Netherlands	news
volkskrant	100064419740547	Netherlands	news
NRC	100064594515527	Netherlands	news
telegraaf	100064754766664	Netherlands	news
AD.NL	100064759384137	Netherlands	news
DGPrawna	100028285615891	Poland	news
gazetaparkiet	100063961094626	Poland	news
dziennikrzeczpospolita	100064275386233	Poland	news

wyborcza	100064301726685	Poland	news
PulsBiznesu	100064747626325	Poland	news
fakt24pl	100064929122435	Poland	news
wwwsepl	100064935690010	Poland	news
vidaeconomica.pt	100063540622545	Portugal	news
impresa.sgps	100063592030034	Portugal	news
JornaldoCentro	100063681337633	Portugal	news
jornaldiabo	100063724835785	Portugal	news
ECOEconomiaOnline	100063888847458	Portugal	news
jornalnegocios	100064315614744	Portugal	news
DiariodeNoticias.pt	100064348103283	Portugal	news
ionline.jornal	100064372072382	Portugal	news
Publico	100064388515461	Portugal	news
abolapt	100064419444773	Portugal	news
jornalnoticias	100064621156693	Portugal	news
ominhopt	100064709201147	Portugal	news
cmjornal	100064843775163	Portugal	news
jornalsol	100064941511794	Portugal	news
ObservadorOnTime	100066638765808	Portugal	news
JornalEconomico	100069127432851	Portugal	news
reportertalequal	100063213640916	Portugal	news
ptjornal	100063749164889	Portugal	news
MoneyBuzzEurope	100035986154156	Romania	news
CursDeGuvernare.ro	100060497983866	Romania	news
romanialibera.ro	100063470753859	Romania	news
kronikaonline.ro	100063626065744	Romania	news
jurnalulnational.ro	100063647073116	Romania	news
BURSAziarul	100063691834070	Romania	news
ziarulCotidianul	100064028379603	Romania	news
libertatea	100064395655471	Romania	news
dcnews.ro	100064582006510	Romania	news
Adevarul	100064603817367	Romania	news
maszol	100064686641712	Romania	news
Gandul.ro	100064818903335	Romania	news
ZIARULFINANCIAR	100071267282074	Romania	news
canescu	100064976672275	Romania	news
ujszo	100048135651928	Slovakia	news
SlovakSpectator	100063772534008	Slovakia	news
pravdadennik	100064531731008	Slovakia	news
novycas	100064654577600	Slovakia	news
sme.sk	100064749578606	Slovakia	news
hospodarskenoviny	100064897321126	Slovakia	news
plusjedenden	100064929392561	Slovakia	news
svet24.si	100063497786519	Slovenia	news
CasnikVecer	100063936340402	Slovenia	news
Finance.si	100064434804227	Slovenia	news
DELO.FB	100064662640626	Slovenia	news
slovenske.novice	100064875963172	Slovenia	news
NoticiasNavarra	100019401653059	Spain	news
lavozdealmeria	100029017286159	Spain	news

diariodesevilla	100047960215028	Spain	news
DiarioDeLeon	100051375938264	Spain	news
eldiacordoba	100063458383497	Spain	news
DiarioLaRioja	100063512145314	Spain	news
diariojaen.es	100063546819940	Spain	news
DiarioCORDOBA.es	100063547600306	Spain	news
diaridetarragona	100063548291173	Spain	news
diariodeburgos	100063560171645	Spain	news
nortecastilla	100063575454204	Spain	news
laopinioncoruna	100063580974266	Spain	news
ElPeriodicoMediterraneo	100063588727758	Spain	news
Diario.de.Pontevedra	100063590739569	Spain	news
eldia.estenerife	100063626318190	Spain	news
opiniondezamora	100063631883187	Spain	news
elprogresodelugo	100063640566751	Spain	news
diariodecadiz	100063684634830	Spain	news
deia.bizkaia	100063703551907	Spain	news
diaridegirona.cat	100063752540145	Spain	news
PeriodicoMalagaHoy	100063759901420	Spain	news
diariodenavarra	100063773761639	Spain	news
diarisegre	100063784614869	Spain	news
HuelvaInformacion	100063788940466	Spain	news
diariregio7	100063847788773	Spain	news
periodicoextremadura	100063856838592	Spain	news
elperiodicodearagon	100063969398724	Spain	news
diariodeibiza	100064326596164	Spain	news
elmundo	100064357437082	Spain	news
eldiariomontanes.es	100064371112029	Spain	news
diariodemallorca.es	100064373426500	Spain	news
diariARA	100064414190436	Spain	news
larazon.es	100064423462353	Spain	news
eldia.es	100064481508714	Spain	news
elperiodico.catalunya	100064481812766	Spain	news
www.ideal.es	100064496359536	Spain	news
lavozdegalicia	100064501791840	Spain	news
heraldodearagon	100064558874508	Spain	news
UltimaHora.Mallorca	100064573876211	Spain	news
elcomercio	100064580178244	Spain	news
diariosur	100064589025484	Spain	news
farodevigo	100064606002951	Spain	news
DiariodeAvisos	100064654545263	Spain	news
ABCes	100064701999354	Spain	news
levante.emv	100064741658917	Spain	news
laopiniondemurcia.es	100064748464354	Spain	news
nuevaespana	100064767338201	Spain	news
LaVanguardia	100064793763427	Spain	news
hoyextremadura	100064840236463	Spain	news
diariolaverdad	100064841526471	Spain	news
diariovascocom	100064855080891	Spain	news
ElCorreo	100064909053219	Spain	news

laprovincia.es	100064917873048	Spain	news
elpais	100064926330075	Spain	news
noticiasdegipuzkoa	100064967100913	Spain	news
periodicoCanarias7	100064983510021	Spain	news
lasprovincias.es	100067782411057	Spain	news
diariodejerez	100080136296223	Spain	news
elpuntavui	100062338941284	Spain	news
dn.se	100064531996146	Sweden	news
svenskadagbladet	100064379186101	Sweden	news
goteborgsposten	100064752218698	Sweden	news
sydsvenskan	100063611334476	Sweden	news
abdebatt	100063678544586	Sweden	news
cityam	100042169882279	UK	news
kidderminstershuttle	100057696345270	UK	news
prospectmagazineuk	100063476833733	UK	news
tribuneuk	100063487873606	UK	news
TheIndependentOnline	100064380085957	UK	news
timesandsundaytimes	100064422172429	UK	news
MetroUK	100064424360523	UK	news
theguardian	100064447883826	UK	news
Spectator1828	100064454629046	UK	news
TheEconomist	100064455893534	UK	news
eveningstandard	100064555755362	UK	news
bbcnews	100064620046507	UK	news
theipaper	100064740424821	UK	news
PrivateEyeNews	100064818427719	UK	news
financialtimes	100064901583665	UK	news
TELEGRAPH.CO.UK	100064939231977	UK	news
NewStatesman	100068572983672	UK	news
EurasiaReview	100032095160838	USA	news
ForeignAffairs	100053276223765	USA	news
Engadget	100053541121821	USA	news
usatoday	100053584873398	USA	news
nytimes	100059174186752	USA	news
HuffPost	100059215554772	USA	news
DailyCaller	100059237543900	USA	news
yahoonews	100059270842861	USA	news
bloombergbusiness	100059283082154	USA	news
WSJ	100059298201442	USA	news
ABCNews	100059306690876	USA	news
politico	100059376258365	USA	news
msnbc	100059391108173	USA	news
FoxNews	100059396147148	USA	news
forbes	100059399986753	USA	news
TheWashingtonTimes	100059401606792	USA	news
CBSNews	100059455603099	USA	news
NBCNews	100059456233501	USA	news
washingtonpost	100059456532991	USA	news
NYPost	100059460163041	USA	news
TheNationMagazine	100059490581690	USA	news

gizmodo	100059494301555	USA	news
techcrunch	100059516290428	USA	news
cnninternational	100059551808655	USA	news
wired	100059568037979	USA	news
usnewsandworldreport	100059571847825	USA	news
WashingtonExaminer	100059597015374	USA	news
NPR	100059610244841	USA	news
theGrio	100059647383948	USA	news
barrons	100059697601796	USA	news
marketwatch	100059709901254	USA	news
cnbc	100059742060475	USA	news
investorsbusinessdaily	100059747820367	USA	news
saltlaketribune	100063490702230	USA	news
sbindependent	100063668253934	USA	news
	100063678920381	USA	news
ladailynews	100063883393276	USA	news
bnonews	100063913991240	USA	news
SiouxCityJournal	100063924098778	USA	news
wcfcourier	100063962653539	USA	news
opednews	100064259872270	USA	news
NYDailyNews	100064372856805	USA	news
triblive	100064381554649	USA	news
SanDiegoUnionTribune	100064390465882	USA	news
daytondailynews	100064454990485	USA	news
chroncom	100064496152443	USA	news
TheBuffaloNews	100064534010740	USA	news
CBSBaltimore	100064551109620	USA	news
DefenseNews	100064562058947	USA	news
HumanRightsWatch	100064565448877	USA	news
globe	100064568716674	USA	news
mercurynews	100064570968638	USA	news
IBTimes	100064580143820	USA	news
seattletimes	100064602019589	USA	news
journalsentinel	100064604477165	USA	news
DesMoinesRegister	100064655502610	USA	news
miamiherald	100064669184922	USA	news
thecharlotteobserver	100064679351790	USA	news
latimes	100064706439366	USA	news
PioneerPress	100064708146763	USA	news
bostonherald	100064727946429	USA	news
dallasmorningnews	100064765418303	USA	news
nrginstitute	100064788966864	USA	news
LincolnJournalStar	100064802798073	USA	news
newsday	100064837746772	USA	news
thechicagosuntimes	100064845483877	USA	news
tampabaycom	100064851515972	USA	news
detroitfreepress	100064864835171	USA	news
SFGate	100064872484667	USA	news
Enquirer	100064912803050	USA	news
startribune	100064921502969	USA	news

denverpost	100064934941984	USA	news
baltimoresun	100064935542026	USA	news
theoregonian	100064955011108	USA	news
staradvertiser	100066454003642	USA	news
PatriotPost	100066570613445	USA	news
MPRnews	100066778755992	USA	news
SaudiGazette	100068922561280	USA	news
WorldHerald	100069228813583	USA	news
ChristianScienceMonitor	100070056773525	USA	news
Emirates247	100070107735954	USA	news
chicagotribune	100071184386238	USA	news
kansascitystar	100076226054459	USA	news
digitaljournal	100063581034088	USA	news
prnewswireglobal	100063678920381	USA	news
volksblatt.at	100045120062884	Austria	parties
neuetageszeitung	100063825434906	Austria	parties
derStandardat	100064428591639	Austria	parties
oe24.at	100064437983860	Austria	parties
TirolerTageszeitung	100064529960707	Austria	parties
DiePressecom	100064626826428	Austria	parties
heute	100064758518481	Austria	parties
salzburger.nachrichten	100064915682949	Austria	parties
kleinezeitung	100064954141289	Austria	parties
KURIER	100071245366349	Austria	parties
degentenaar	100048634435435	Belgium	parties
detijd	100063616747864	Belgium	parties
metrobelgique	100063718684113	Belgium	parties
GRENZECHO.net	100064316070895	Belgium	parties
hetbelangvanlimburg	100064480522666	Belgium	parties
demorgen	100064499871779	Belgium	parties
lesoirbe	100064530170861	Belgium	parties
nieuwsblad.be	100064557829768	Belgium	parties
lavenir.net	100064593223358	Belgium	parties
lalibre.be	100064623613822	Belgium	parties
destandaard	100064703858214	Belgium	parties
BrusselsTimes	100064717417376	Belgium	parties
Gazetvanantwerpen	100064773730560	Belgium	parties
lechobe	100064868255048	Belgium	parties
telegraph.bg	100063585639566	Bulgaria	parties
borbavt	100063679266749	Bulgaria	parties
trud.bg	100063938644879	Bulgaria	parties
24chasa	100064254836629	Bulgaria	parties
sega.bulgaria	100064521319288	Bulgaria	parties
vestnikbanker	100064528636494	Bulgaria	parties
standartnews	100064653135465	Bulgaria	parties
dnevnik	100064734488972	Bulgaria	parties
dnesbulgaria	100083147509857	Bulgaria	parties
montpress	100048912222554	Bulgaria	parties
Glasistre.hr	100028989566235	Croatia	parties
Glas.Slavonije	100056653586924	Croatia	parties

novilist	100063474323974	Croatia	parties
zadarski.hr	100063678693713	Croatia	parties
Dubrovackivjesnik	100063707313492	Croatia	parties
poslovni.hr	100064380113366	Croatia	parties
vecernji	100064521411238	Croatia	parties
slobodnadalmacija	100064622562165	Croatia	parties
jutarnji.list	100064691530707	Croatia	parties
Haravgi	100050317346041	Cyprus	parties
simerini.news	100063526965440	Cyprus	parties
politisnewspaper	100063639114710	Cyprus	parties
AlithiaMedia	100063679384921	Cyprus	parties
CyprusMail	100063685807707	Cyprus	parties
phileleftheros	100064363402618	Cyprus	parties
cynewseu	100064818153196	Cyprus	parties
denik.cz	100063539801434	Czech	parties
denikpravo	100063543933911	Czech	parties
Blesk.cz	100064364576701	Czech	parties
hospodarky	100064371956072	Czech	parties
MladaFrontaDNES	100063855720670	Czech	parties
lidovenoviny	100057511403915	Czech	parties
copenhagenpost	100052648913632	Denmark	parties
arbejderen	100057062757080	Denmark	parties
Weekendavisen	100063478336171	Denmark	parties
kristeligt	100063617634377	Denmark	parties
berlingske	100064331127072	Denmark	parties
ekstrabladet	100064630966309	Denmark	parties
jyllandsposten	100064681511805	Denmark	parties
borsen.dk	100064732057174	Denmark	parties
politiken	100064763048362	Denmark	parties
ditbt	100064835255015	Denmark	parties
dagbladetinformation	100064985370038	Denmark	parties
saartehaal	100060830390957	Estonia	parties
aripaev	100063516791933	Estonia	parties
eestipaevaleht	100063559421999	Estonia	parties
postimees	100064361962471	Estonia	parties
Ohtuleht	100064573758796	Estonia	parties
helsinginsanomat	100064708989284	Finland	parties
Hufvudstadsbladet	100063691744768	Finland	parties
esaimaa	100063675423295	Finland	parties
lansisuomi	100063279017323	Finland	parties
ksmlfi	100046602140170	Finland	parties
KalevaOy	100064052225054	Finland	parties
vasabladet.fi	100063762784502	Finland	parties
HameenSanomat	100064209157034	Finland	parties
savonsanomat1	100064139465052	Finland	parties
kainuunsanomat.fi	100034431856916	Finland	parties
	100043574843659	Finland	parties
lansiuusimaa	100043967245661	Finland	parties
satakunnankansa	100029619590706	Finland	parties
aamulehti	100058005080883	Finland	parties

salonseudunsanomat	100063611108579	Finland	parties
Ilkkapohjalainen.fi	100038120417469	Finland	parties
lapinkansa	100063761552409	Finland	parties
uusimaa.fi	100063707643218	Finland	parties
iisalmensanomat	100064143879954	Finland	parties
kauppalehti.fi	100063543011493	Finland	parties
abounderrattelser	100063771992702	Finland	parties
taloussanomat	100063640656805	Finland	parties
kouvolansanomat	100063610268661	Finland	parties
nyaaland	100041521376428	Finland	parties
lansisavo	100049970485384	Finland	parties
turunsanomat	100063645114913	Finland	parties
kymensanomat	100063593829272	Finland	parties
SanomalehtiKarjalainen	100023966306947	Finland	parties
Keskilaakso	100063753334909	Finland	parties
osterbottenstidning	100063702543765	Finland	parties
keskipohjanmaa	100063631387875	Finland	parties
kansanuutiset	100041901475678	Finland	parties
aamuposti	100063626048399	Finland	parties
demokraatti.fi	100063032390600	Finland	parties
alandstidningen	100063661385369	Finland	parties
itahame	100028107566237	Finland	parties
itasavo	100041220175772	Finland	parties
vastranyland	100040087584524	Finland	parties
tyrvaansanomat	100053561535865	Finland	parties
KarjalanH	100063548770229	Finland	parties
lequipe.fr	100044348443220	France	parties
Contrepoints	100063727893534	France	parties
lacroix.journal	100063778574382	France	parties
lemonde.fr	100064539140308	France	parties
lefigaro	100064714449291	France	parties
lesechos	100064763164049	France	parties
humanite.fr	100064860845478	France	parties
Mediapart.fr	100064909983213	France	parties
20minutes	100064974150667	France	parties
Liberation	100064986330109	France	parties
leparisien	100069002424655	France	parties
latribune	100078075303669	France	parties
junge.welt	100046632146318	Germany	parties
welt	100053495553917	Germany	parties
wznewsline	100060895134337	Germany	parties
FrankfurterRundschau	100063650589063	Germany	parties
nd.aktuell	100063791130047	Germany	parties
rponline	100064293565184	Germany	parties
berlinerzeitung	100064302865032	Germany	parties
taz.kommune	100064324914179	Germany	parties
bild	100064507341597	Germany	parties
Tagesspiegel	100064522520895	Germany	parties
faz	100064567848864	Germany	parties
handelsblatt	100064653795335	Germany	parties

derspiegel	100064772348380	Germany	parties
ZDFheute	100064809428075	Germany	parties
zeitonline	100064829557159	Germany	parties
ihre.sz	100064830484979	Germany	parties
stern	100064869632404	Germany	parties
DromosThsAristeras	100062329604090	Greece	parties
tomanifesto.gr	100063536504637	Greece	parties
AvgiOnline	100063621801820	Greece	parties
eleftheriora	100063696652610	Greece	parties
vradini.gr	100063734023729	Greece	parties
karfitsa.gr	100063749525212	Greece	parties
dimokratianews.gr	100063791012388	Greece	parties
paron.gr	100063872528301	Greece	parties
EleftherosTypos	100064014158824	Greece	parties
newsmakedonias	100064023874001	Greece	parties
efmpam	100064095483038	Greece	parties
pringr1	100064107127357	Greece	parties
paraskhnio	100064119580269	Greece	parties
taneagr	100064440201946	Greece	parties
www.parapolitika.gr	100064496034215	Greece	parties
efimerida.syntakton	100064539228165	Greece	parties
topontiki	100064968360805	Greece	parties
valaszonline	100032983166134	Hungary	parties
UngarnHeute	100032983640728	Hungary	parties
dailynewshungary	100039554211314	Hungary	parties
vasarnap.hu	100043995405628	Hungary	parties
magyarkurir	100047641015458	Hungary	parties
Metropol.Napilap	100052786192503	Hungary	parties
telexhu	100054073007151	Hungary	parties
media1hu	100063327212183	Hungary	parties
BudapesterZeitung	100063567550956	Hungary	parties
magyarnemzet.hu	100063619687797	Hungary	parties
nepszavaonline	100063643492521	Hungary	parties
magyarjelen	100063746605581	Hungary	parties
pestibulvar	100063773312676	Hungary	parties
velvethu	100063912728962	Hungary	parties
ezalenyeg.hu	100064082855223	Hungary	parties
magyarinfo	100064324014009	Hungary	parties
24ponthu	100064331216521	Hungary	parties
Borsonline.Bors.Szorakoztato.Napilap	100064364726814	Hungary	parties
AlfahirHirportal	100064516427295	Hungary	parties
economx.hu	100064544927214	Hungary	parties
Indexhu	100064585093978	Hungary	parties
portfolio.hu	100064669694898	Hungary	parties
168ora	100064672989973	Hungary	parties
hungarytoday	100064734217203	Hungary	parties
blikkhu	100064763644053	Hungary	parties
444.hu	100064881090307	Hungary	parties
atlatszo.hu	100067717692359	Hungary	parties
AboutHungary	100069124123415	Hungary	parties

OrigoHirek	100069355143217	Hungary	parties
magyarhirlapfb	61550744906230	Hungary	parties
kitekinto	100034708220685	Hungary	parties
journalfrancophonedebudapest	100036175584070	Hungary	parties
dailythanthi	100064236057276	India	parties
mathrubhumidotcom	100064275505737	India	parties
Madhyamam	100064321164121	India	parties
patrikahindinews	100064489187935	India	parties
hindustantimes	100064660902473	India	parties
AnandabazarSocial	100064721134943	India	parties
Sakshinews	100064739974516	India	parties
manoramaonline	100064748133924	India	parties
Pkesarionline	100064770070599	India	parties
enavabharat	100064778105936	India	parties
TheNavbharatlive	100064778105936	India	parties
DailyGreaterKashmir	100064778583465	India	parties
DeepikaNewspaper	100064883342294	India	parties
sirajonlive	100064934820717	India	parties
sangbadpratidin.in	100066353026637	India	parties
thehindu	100069641816229	India	parties
eenaduonline	100070918585406	India	parties
DinamalarDistrictNews	100076824197305	India	parties
lokmat	100077795915005	India	parties
IrishHerald	100063540608271	Ireland	parties
IrishDailyStar	100063793292361	Ireland	parties
theirishsun	100064242387150	Ireland	parties
irishmirror	100064380833692	Ireland	parties
irishtimes	100064447823624	Ireland	parties
IrishExaminer	100064837142381	Ireland	parties
Independent.ie	100064865345158	Ireland	parties
domanigiornale	100039815302405	Italy	parties
tgla7	100046840735192	Italy	parties
ilFattoQuotidiano	100053429766843	Italy	parties
Ildubbionews	100063545110411	Italy	parties
milanofinanza	100063625867727	Italy	parties
ilsole24ore	100064360407072	Italy	parties
corrieredellasera	100064368746696	Italy	parties
SkyTG24	100064554647327	Italy	parties
liberonews	100064555695145	Italy	parties
AgenziaANSA	100064615457043	Italy	parties
fanpage.it	100064653281114	Italy	parties
ilmanifesto	100064667114396	Italy	parties
quotidianolaverita	100064678177949	Italy	parties
tgcom24	100064680279179	Italy	parties
avvenire.it	100064688078819	Italy	parties
RaiNews	100064695876217	Italy	parties
ilfoglio	100064802288107	Italy	parties
ilGiornale	100064829017156	Italy	parties
Repubblica	100070283104562	Italy	parties
lastampa.it	100071317846404	Italy	parties

DienaLV	100046659913828	Latvia	parties
DienasBizness	100057396762122	Latvia	parties
wwwIRlv	100063579130070	Latvia	parties
LatvijasAvize	100063777901867	Latvia	parties
nralv	100064124948564	Latvia	parties
LatvijasVestnesis	100064738241163	Latvia	parties
verslozinios	100063528673426	Lithuania	parties
respublika.lt	100063770723844	Lithuania	parties
lrytaslt	100064693330778	Lithuania	parties
TheLithuaniaTribune	100060215265123	Lithuania	parties
telecran.luxemburg	100044189815158	Luxemburg	parties
Tageblatt.lu	100046508845546	Luxemburg	parties
woxx.lu	100062610230174	Luxemburg	parties
revue.lu	100063003290239	Luxemburg	parties
	100063581950772	Luxemburg	parties
wort.lu.de	100063889300522	Luxemburg	parties
letzjournal	100063949338917	Luxemburg	parties
LeQuotidien.lu	100063965316913	Luxemburg	parties
brozlv	100065158352515	Luxemburg	parties
lessentielonline	100067298233955	Luxemburg	parties
goosch.lu	100063951463477	Luxemburg	parties
IllumMediaToday	100031468608077	Malta	parties
TheMaltaIndependent	100064088889987	Malta	parties
NetNewsMalta	100064732508964	Malta	parties
ONE.com.mt	100064779873833	Malta	parties
timesofmalta	100064984770069	Malta	parties
Cuartopodermx	100058908474479	Mexico	parties
cambiodemichoacan	100063463493908	Mexico	parties
periodicoelautonomo	100063920115779	Mexico	parties
	100063934808025	Mexico	parties
CampecheHOYmx	100064069412021	Mexico	parties
periodicoeldebate	100064293504836	Mexico	parties
elimparcialcom	100064370546242	Mexico	parties
MilenioDiario	100064424424683	Mexico	parties
criteriohidalgo	100064456642032	Mexico	parties
ElDiarioDelYaqui	100064459191590	Mexico	parties
lajornadaonline	100064499151846	Mexico	parties
ElUniversalOnline	100064562299062	Mexico	parties
LaCronicaDeHoy	100064643085866	Mexico	parties
abcnoticiasmty	100064728754683	Mexico	parties
revistaproceso	100064917032757	Mexico	parties
ExcelsiorMex	100064918140477	Mexico	parties
ElEconomista.mx	100064918230835	Mexico	parties
elinformador	100067238112404	Mexico	parties
DiariodeYucatan	100070081782559	Mexico	parties
elheraldodemexico	100071338323935	Mexico	parties
eldebatecom	100076403791728	Mexico	parties
	100083883133633	Mexico	parties
	100031928451274	Mexico	parties
Trouw.nl	100063462223346	Netherlands	parties

refdag	100063773238966	Netherlands	parties
het.financieele.dagblad	100063973664746	Netherlands	parties
nederlandsdagblad	100064118551566	Netherlands	parties
volkskrant	100064419740547	Netherlands	parties
NRC	100064594515527	Netherlands	parties
telegraaf	100064754766664	Netherlands	parties
AD.NL	100064759384137	Netherlands	parties
DGPrawna	100028285615891	Poland	parties
gazetaparkiet	100063961094626	Poland	parties
dziennikrzeczpospolita	100064275386233	Poland	parties
wyborcza	100064301726685	Poland	parties
PulsBiznesu	100064747626325	Poland	parties
fakt24pl	100064929122435	Poland	parties
wwwsepl	100064935690010	Poland	parties
vidaeconomica.pt	100063540622545	Portugal	parties
impresa.sgps	100063592030034	Portugal	parties
JornaldoCentro	100063681337633	Portugal	parties
jornaldiabo	100063724835785	Portugal	parties
ECOEconomiaOnline	100063888847458	Portugal	parties
jornalnegocios	100064315614744	Portugal	parties
DiariodeNoticias.pt	100064348103283	Portugal	parties
ionline.jornal	100064372072382	Portugal	parties
Publico	100064388515461	Portugal	parties
abolapt	100064419444773	Portugal	parties
jornalnoticias	100064621156693	Portugal	parties
ominhopt	100064709201147	Portugal	parties
cmjornal	100064843775163	Portugal	parties
jornalsol	100064941511794	Portugal	parties
ObservadorOnTime	100066638765808	Portugal	parties
JornalEconomico	100069127432851	Portugal	parties
reportertalequal	100063213640916	Portugal	parties
ptjornal	100063749164889	Portugal	parties
MoneyBuzzEurope	100035986154156	Romania	parties
CursDeGuvernare.ro	100060497983866	Romania	parties
romanialibera.ro	100063470753859	Romania	parties
kronikaonline.ro	100063626065744	Romania	parties
jurnalulnational.ro	100063647073116	Romania	parties
BURSAziarul	100063691834070	Romania	parties
ziarulCotidianul	100064028379603	Romania	parties
libertatea	100064395655471	Romania	parties
dcnews.ro	100064582006510	Romania	parties
Adevarul	100064603817367	Romania	parties
maszol	100064686641712	Romania	parties
Gandul.ro	100064818903335	Romania	parties
ZIARULFINANCIAR	100071267282074	Romania	parties
canescu	100064976672275	Romania	parties
ujszo	100048135651928	Slovakia	parties
SlovakSpectator	100063772534008	Slovakia	parties
pravdadennik	100064531731008	Slovakia	parties
novycas	100064654577600	Slovakia	parties

sme.sk	100064749578606	Slovakia	parties
hospodarskenoviny	100064897321126	Slovakia	parties
plusjedenden	100064929392561	Slovakia	parties
svet24.si	100063497786519	Slovenia	parties
CasnikVecer	100063936340402	Slovenia	parties
Finance.si	100064434804227	Slovenia	parties
DELO.FB	100064662640626	Slovenia	parties
slovenske.novice	100064875963172	Slovenia	parties
NoticiasNavarra	100019401653059	Spain	parties
lavozdealmeria	100029017286159	Spain	parties
diariodesevilla	100047960215028	Spain	parties
DiarioDeLeon	100051375938264	Spain	parties
eldiacordoba	100063458383497	Spain	parties
DiarioLaRioja	100063512145314	Spain	parties
diariojaen.es	100063546819940	Spain	parties
DiarioCORDOBA.es	100063547600306	Spain	parties
diaridetarragona	100063548291173	Spain	parties
diariodeburgos	100063560171645	Spain	parties
nortecastilla	100063575454204	Spain	parties
laopinioncoruna	100063580974266	Spain	parties
ElPeriodicoMediterraneo	100063588727758	Spain	parties
Diario.de.Pontevedra	100063590739569	Spain	parties
eldia.estenerife	100063626318190	Spain	parties
opiniondezamora	100063631883187	Spain	parties
elprogresodelugo	100063640566751	Spain	parties
diariodecadiz	100063684634830	Spain	parties
deia.bizkaia	100063703551907	Spain	parties
diaridegirona.cat	100063752540145	Spain	parties
PeriodicoMalagaHoy	100063759901420	Spain	parties
diariodenavarra	100063773761639	Spain	parties
diarisegre	100063784614869	Spain	parties
HuelvaInformacion	100063788940466	Spain	parties
diariregio7	100063847788773	Spain	parties
periodicoextremadura	100063856838592	Spain	parties
elperiodicodearagon	100063969398724	Spain	parties
diariodeibiza	100064326596164	Spain	parties
elmundo	100064357437082	Spain	parties
eldiariomontanes.es	100064371112029	Spain	parties
diariodemallorca.es	100064373426500	Spain	parties
diariARA	100064414190436	Spain	parties
larazon.es	100064423462353	Spain	parties
eldia.es	100064481508714	Spain	parties
elperiodico.catalunya	100064481812766	Spain	parties
www.ideal.es	100064496359536	Spain	parties
lavozdegalicia	100064501791840	Spain	parties
heraldodearagon	100064558874508	Spain	parties
UltimaHora.Mallorca	100064573876211	Spain	parties
elcomercio	100064580178244	Spain	parties
diariosur	100064589025484	Spain	parties
farodevigo	100064606002951	Spain	parties

DiariodeAvisos	100064654545263	Spain	parties
ABCes	100064701999354	Spain	parties
levante.emv	100064741658917	Spain	parties
laopiniondemurcia.es	100064748464354	Spain	parties
nuevaespana	100064767338201	Spain	parties
LaVanguardia	100064793763427	Spain	parties
hoyextremadura	100064840236463	Spain	parties
diariolaverdad	100064841526471	Spain	parties
diariovascocom	100064855080891	Spain	parties
ElCorreo	100064909053219	Spain	parties
laprovincia.es	100064917873048	Spain	parties
elpais	100064926330075	Spain	parties
noticiasdegipuzkoa	100064967100913	Spain	parties
periodicoCanarias7	100064983510021	Spain	parties
lasprovincias.es	100067782411057	Spain	parties
diariodejerez	100080136296223	Spain	parties
elpuntavui	100062338941284	Spain	parties
dn.se	100064531996146	Sweden	parties
svenskadagbladet	100064379186101	Sweden	parties
goteborgsposten	100064752218698	Sweden	parties
sydsvenskan	100063611334476	Sweden	parties
abdebatt	100063678544586	Sweden	parties
cityam	100042169882279	UK	parties
kidderminstershuttle	100057696345270	UK	parties
prospectmagazineuk	100063476833733	UK	parties
tribuneuk	100063487873606	UK	parties
TheIndependentOnline	100064380085957	UK	parties
timesandsundaytimes	100064422172429	UK	parties
MetroUK	100064424360523	UK	parties
theguardian	100064447883826	UK	parties
Spectator1828	100064454629046	UK	parties
TheEconomist	100064455893534	UK	parties
eveningstandard	100064555755362	UK	parties
bbcnews	100064620046507	UK	parties
theipaper	100064740424821	UK	parties
PrivateEyeNews	100064818427719	UK	parties
financialtimes	100064901583665	UK	parties
TELEGRAPH.CO.UK	100064939231977	UK	parties
NewStatesman	100068572983672	UK	parties
EurasiaReview	100032095160838	USA	parties
ForeignAffairs	100053276223765	USA	parties
Engadget	100053541121821	USA	parties
usatoday	100053584873398	USA	parties
nytimes	100059174186752	USA	parties
HuffPost	100059215554772	USA	parties
DailyCaller	100059237543900	USA	parties
yahoonews	100059270842861	USA	parties
bloombergbusiness	100059283082154	USA	parties
WSJ	100059298201442	USA	parties
ABCNews	100059306690876	USA	parties

politico	100059376258365	USA	parties
msnbc	100059391108173	USA	parties
FoxNews	100059396147148	USA	parties
forbes	100059399986753	USA	parties
TheWashingtonTimes	100059401606792	USA	parties
CBSNews	100059455603099	USA	parties
NBCNews	100059456233501	USA	parties
washingtonpost	100059456532991	USA	parties
NYPost	100059460163041	USA	parties
TheNationMagazine	100059490581690	USA	parties
gizmodo	100059494301555	USA	parties
techcrunch	100059516290428	USA	parties
cnninternational	100059551808655	USA	parties
wired	100059568037979	USA	parties
usnewsandworldreport	100059571847825	USA	parties
WashingtonExaminer	100059597015374	USA	parties
NPR	100059610244841	USA	parties
theGrio	100059647383948	USA	parties
barrons	100059697601796	USA	parties
marketwatch	100059709901254	USA	parties
cnbc	100059742060475	USA	parties
investorsbusinessdaily	100059747820367	USA	parties
saltlaketribune	100063490702230	USA	parties
sbindependent	100063668253934	USA	parties
	100063678920381	USA	parties
ladailynews	100063883393276	USA	parties
bnonews	100063913991240	USA	parties
SiouxCityJournal	100063924098778	USA	parties
wcfcourier	100063962653539	USA	parties
opednews	100064259872270	USA	parties
NYDailyNews	100064372856805	USA	parties
triblive	100064381554649	USA	parties
SanDiegoUnionTribune	100064390465882	USA	parties
daytondailynews	100064454990485	USA	parties
chroncom	100064496152443	USA	parties
TheBuffaloNews	100064534010740	USA	parties
CBSBaltimore	100064551109620	USA	parties
DefenseNews	100064562058947	USA	parties
HumanRightsWatch	100064565448877	USA	parties
globe	100064568716674	USA	parties
mercurynews	100064570968638	USA	parties
IBTimes	100064580143820	USA	parties
seattletimes	100064602019589	USA	parties
journalsentinel	100064604477165	USA	parties
DesMoinesRegister	100064655502610	USA	parties
miamiherald	100064669184922	USA	parties
thecharlotteobserver	100064679351790	USA	parties
latimes	100064706439366	USA	parties
PioneerPress	100064708146763	USA	parties
bostonherald	100064727946429	USA	parties

dallasmorningnews	100064765418303	USA	parties
nrginstitute	100064788966864	USA	parties
LincolnJournalStar	100064802798073	USA	parties
newsday	100064837746772	USA	parties
thechicagosuntimes	100064845483877	USA	parties
tampabaycom	100064851515972	USA	parties
detroitfreepress	100064864835171	USA	parties
SFGate	100064872484667	USA	parties
Enquirer	100064912803050	USA	parties
startribune	100064921502969	USA	parties
denverpost	100064934941984	USA	parties
baltimoresun	100064935542026	USA	parties
theoregonian	100064955011108	USA	parties
staradvertiser	100066454003642	USA	parties
PatriotPost	100066570613445	USA	parties
MPRnews	100066778755992	USA	parties
SaudiGazette	100068922561280	USA	parties
WorldHerald	100069228813583	USA	parties
ChristianScienceMonitor	100070056773525	USA	parties
Emirates247	100070107735954	USA	parties
chicagotribune	100071184386238	USA	parties
kansascitystar	100076226054459	USA	parties
digitaljournal	100063581034088	USA	parties
prnewswireglobal	100063678920381	USA	parties
