# Supplementary material for: Mapping the global election landscape on social media in 2024
Source: PLoS One. 2025 Feb 5;20(2):e0316271. doi: 10.1371/journal.pone.0316271 (PMC11798462; doi:10.1371/journal.pone.0316271)
Supplement: S2 Table — (PDF) [file pone.0316271.s011.pdf]

Supporting information

Table 1. A sample of posts assigned to the first cluster by the LDA model and the BERTopic-based topic modeling.

LDA_topic	post_text	BERT_based_topic
t_1	the blustery conditions are a stark contrast following a hot weekend in the capital	climate
t_1	a propalestine protest calling for a ceasefire in the israelhamas war will march through the streets of central london today after the uk abstained on a un resolution on the matter	protest
t_1	the finest character actor of his generation passed away a decade ago these are the most exceptional performances he left us with	entertainment
t_1	the fact that attention on the book a book that covers many subjects in gritty detail seems solely focused on my old lady sexual awakening and the younger lover is utterly bemusing stacey duguid	unclear

t_1	winston churchill would be appalled at how the european convention on human rights allows migrants to abuse the asylum system the immigration minister said on tuesday	migration
t_1	in the new netflix drama one day we are rooting for dexter not emma as the vulnerable romantic hero reflecting a changing narrative around gender and relationships read more	gender
t_1	were completely disapplying all the relevant sections of the human rights act rishi sunak confirms that the rwanda plan will block challenges from domestic or international law	eu_politics
t_1	former ukip leader speaks on the russian invasion of ukraine whether he would join the tories and how reform uk will fare	ukraine
t_1	one controversial scene even had to be reedited for the films uk release at the beginning of january	economy

t_1	breaking news us investors are paying the biggest premiums since october to protect their portfolios against market gyrations as tensions mount in the middle east	mena
